# Which modification for which feedstock? a strategy-by-strategy review of Zr-MOF catalysts for biodiesel production

**DOI:** 10.1039/d6ra06445k

**Published:** 2026-09-03

**Authors:** Basem E. Keshta, Eida S. Al-Farraj, Fatimah Bukola Shittu, Shweta Vyas, Lobna A. Heikal, Yasmeen G. Abou El-Reash, Hany Koheil, Yuanbin Zhang

**Affiliations:** a Key Laboratory of Ministry of Education for Advanced Catalysis Materials, Zhejiang Normal University Jinhua 321004 P.R. China b.keshta@zjnu.edu.cn ybzhang@zjnu.edu.cn; b Chemistry Department, Faculty of Science, Tanta University Tanta 31527 Egypt basem.keshta@science.tanta.edu.eg; c Department of Chemistry, College of Science, Imam Mohammad Ibn Saud Islamic University (IMSIU) Riyadh P.O. Box, 90950 11623 Saudi Arabia; d The Federal Polytechnic Offa PMB 420 Kwara State Nigeria; e Department of Pure & Applied Chemistry, University of Kota Kota Rajasthan- 324005 India; f Renewable Energy Science and Engineering Department, Faculty of Postgraduate Studies for Advanced Sciences, Beni-Suef University Beni-Suef 62521 Egypt; g Physics and Engineering Mathematics Department, Faculty of Engineering, Kafr Elsheikh University Kafr Elsheikh 33516 Egypt

## Abstract

Catalyst design is central to producing sustainable biodiesel from waste feedstocks. Homogeneous bases such as NaOH and KOH are highly active but cause corrosion, saponification, and difficult separation, while conventional heterogeneous catalysts suffer from low surface area and poor tunability. Zirconium-based metal–organic frameworks (Zr-MOFs) such as UiO-66 have emerged as attractive alternatives, combining high porosity, exceptional chemical and thermal stability, and tunable Lewis and Brønsted acidity. Pristine Zr-MOFs, however, are limited by low acid site density and by mass transfer resistance toward bulky triglycerides. This review assesses five structural modification strategies reported: metal and node defect engineering, pore engineering, linker and ligand functionalization, composite formation, and enzyme immobilization and, unlike previous surveys, compares them directly against one another rather than in isolation. Each strategy is evaluated on yield, reaction severity, reusability, and tolerance to water and free fatty acids, and matched to the feedstock class it is best suited to address. We show that the central constraint on catalyst design is a trade-off between acid site density and pore accessibility, and that the highest-performing catalysts are those that decouple the two. We further show that only about a third of the studies surveyed test genuine triglyceride transesterification; the remainder use isolated fatty acids or non-lipid model reactions such as benzaldehyde acetalization, levulinic acid conversion, and short-chain ester synthesis, leading to a systematic overstatement of catalytic performance in such literature. Future directions and the challenges of zirconium cost, long-term stability, and scalable green synthesis are discussed.

## Abstract 2

A decision map for Zr-MOF biodiesel catalysts: match the modification to the feedstock, not the trend.

## Graphical abstract

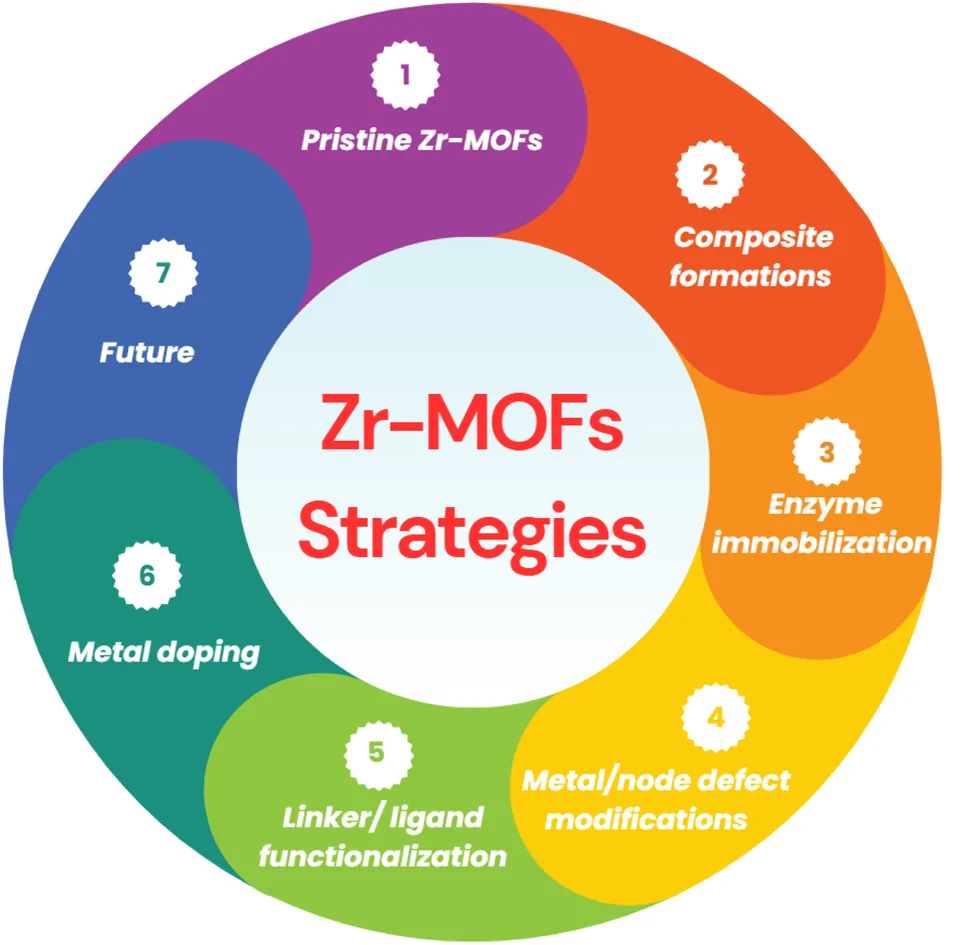

## 1 Introduction

The transport sector accounts for roughly one-quarter (∼23%) of global energy-related CO_2_ emissions and about 15% of total anthropogenic greenhouse gas emissions, while its reliance on petroleum fuels contributes to resource depletion, price instability, and geopolitical dependence.^1–4^ Compared to conventional diesel, biodiesel has a higher cetane number and flash point and provides a sustainable, non-toxic, sulfur-free alternative.^5–7^ Global biodiesel output has grown steadily over the past two decades, with FAME biodiesel production approaching 50 billion litres (∼44 million tonnes) in 2024; Indonesia (∼13 billion L), the European Union (∼11 billion L), Brazil (∼9 billion L), and the United States are currently the dominant producing regions.^5,8–11^ Using homogeneous or heterogeneous catalysts, free fatty acids and triglycerides are esterified or transesterified to create biodiesel.^12^ Two reactions produce biodiesel. In transesterification, a triglyceride reacts with a short-chain alcohol, typically methanol, over an acid or base catalyst to give three molecules of fatty acid methyl ester (FAME) and one of glycerol; the reaction proceeds stepwise through diglyceride and monoglyceride intermediates and is reversible, so excess alcohol is used to drive it forward.^13^ In esterification, a free fatty acid reacts with the alcohol to give the corresponding methyl ester and water and requires an acid catalyst.^14^ The distinction governs catalyst choice. Refined oils are low in free fatty acids and can be transesterified directly, but waste and non-edible feedstocks carry high free fatty acid contents that saponify under basic conditions, so these require an acid catalyst able to esterify the free fatty acids and transesterify the triglycerides at the same time. The choice of catalyst is therefore dictated by the free fatty acid content of the feedstock.^15^ Although homogeneous bases, such as NaOH and KOH, have high activity, they have problems with corrosion, separation, and wastewater production.^16^ Although they provide simpler recovery and reusability, heterogeneous catalysts such metal oxides, ion-exchange resins, and hydrotalcites are constrained by their low surface area and poor tunability.^17,18^

The tunable porosity, large surface area, and functionalize organic linkers of metal–organic frameworks (MOFs) made them versatile platforms across adsorption, separation, and catalysis, where the same structural tunability that governs guest capture and molecular separation also allows active sites to be engineered by design.^15,19–23^ Among these, under severe biodiesel reaction conditions, zirconium-based MOFs (Zr-MOFs) like UiO-66 show remarkable heat and chemical resilience.^24,25^ Lewis acidity is provided by their Zr_6_-oxo clusters, while Brønsted sites arise from terminal hydroxyls at the node and can be introduced further through functionalised linkers.^26,27^ This combination is what makes Zr-MOFs attractive for low-grade feedstocks: a single solid can esterify the free fatty acids and transesterify the triglycerides simultaneously, avoiding the two-step acid pre-treatment that base catalysts require. Reactant adsorption is made easier by high surface-to-volume ratios, and outstanding stability permits 5 to 8 reuse cycles with little activity loss.^28–30^

**Fig. 1:**
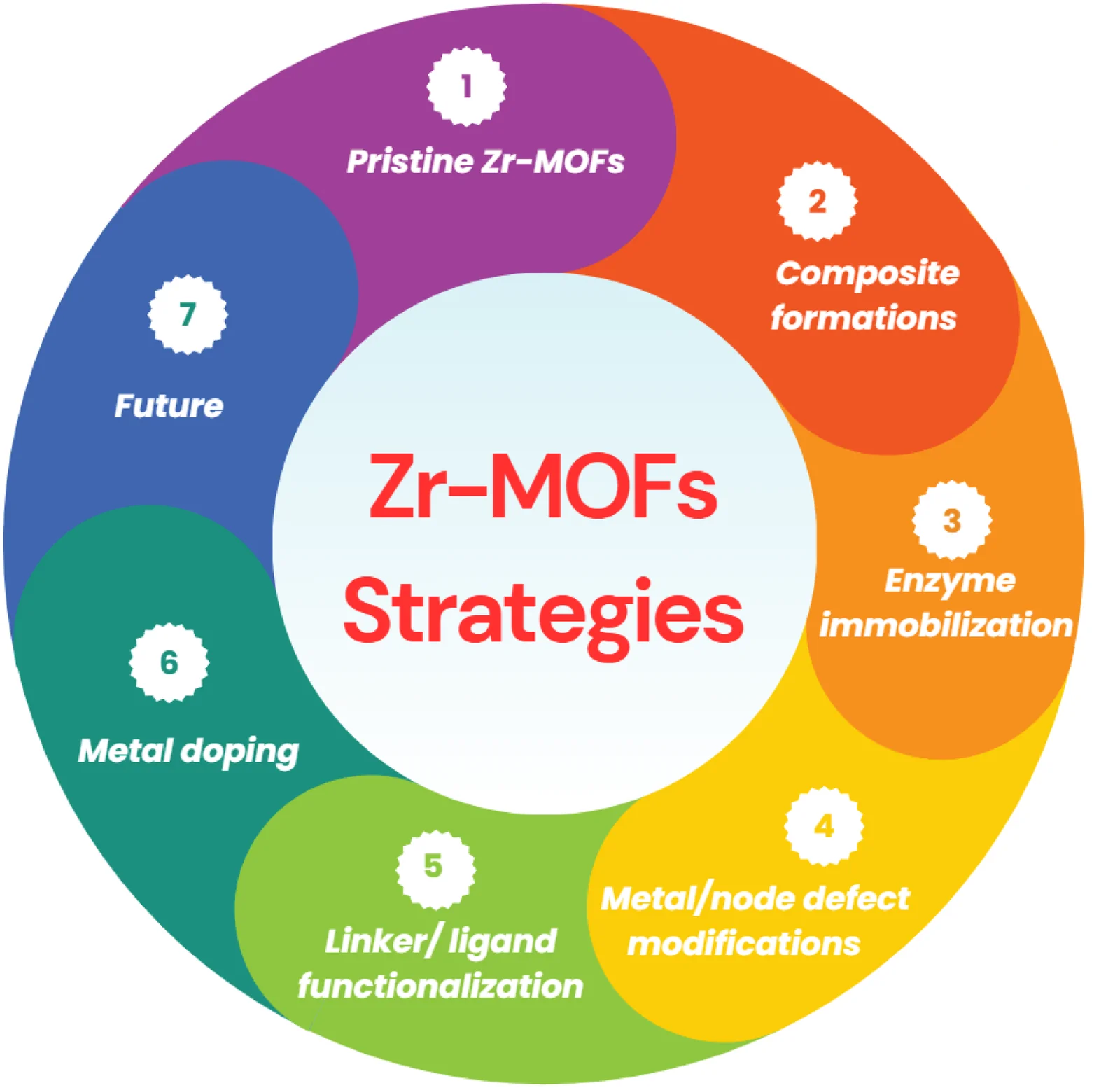
Schematic illustration of Zr-MOF modification strategies for high-efficiency biodiesel production.

Existing reviews, however, cover this space only partially. Broader surveys treat MOF catalysts for biodiesel as a class without isolating the Zr-based subset, while the few dedicated Zr-MOF treatments are brief and organized by application rather than by modification strategy.^15,26,31–34^ What is still missing is a strategy-by-strategy comparison in which the individual structural modification routes are assessed against one another under a common set of performance criteria. The aim of the present review is to address that gap. We critically evaluate five structural modification strategies (Fig. 1) for Zr-MOF catalysts in biodiesel synthesis reported between 2018 and 2026- metal/node defect engineering, pore engineering, linker/ligand functionalization, composite formation, and enzyme immobilization and compare them on catalytic yield, reaction severity, catalyst loading, reusability, and tolerance to water and free fatty acids in low-grade waste feedstocks. For each strategy we link the structural change to the resulting acid-site density, pore accessibility, and framework stability, so that the structure–activity relationships governing performance become explicit rather than anecdotal. It should be noted that Zr-MOFs also serve as platforms for photocatalysis and electrocatalysis in energy and environmental applications, exploiting photoactive linkers and redox-active Zr nodes; biodiesel production, however, rests primarily on acid–base chemistry at Zr^4+^ sites, and this review therefore focuses on acid–base catalysis while acknowledging the broader catalytic versatility of these frameworks.

## 2 Modification strategies of Zr-MOFs for biodiesel production

MOFs are crystalline porous structures with large surface area and configurable functionality for multiphase catalysis that are made up of metal nodes coordinated to organic linkers.^35,36^ Because of their remarkable thermal and chemical stability, high density of Lewis/Brønsted acid sites, and outstanding reusability under challenging transesterification conditions, Zr-MOFs like UiO-66 are particularly appealing among MOFs for biodiesel synthesis.^37^ To overcome mass transfer constraints and improve catalytic performance for the generation of biodiesel, this section addresses typical modification techniques done to virgin Zr-MOFs. Table 1 summarizes the catalytic performance, reaction conditions, and main conclusions of key findings.

**Table 1:** Recent findings on biodiesel production using Zr-based MOF catalysts

Zr-MOFs catalyst	Feedstock	Reaction type	Reaction conditions	Biodiesel conversion/yield (%)	Reusability cycles	Strategy	Ref.
UiO-66(Zr)-solvent	Oleic acid	FFA esterification	60 °C, 4 h, 39 : 1 MeOH, 6 wt%	40	—	Pristine baseline	41
Ca^2+^-doped UiO-66(Zr)	Oleic acid	FFA esterification	60 °C	98	5	Metal defects	43
MOF-808-3/1	Microalgal lipids	Transesterification	200 °C, 4.0 MPa, 20 : 1 MeOH: oil, 2 wt%, 1 h	96.2	6	Pore engineering, metal defects	55
Zr/Ce-MOF-808	Microalgal lipids	Transesterification	100 °C, 0.35 MPa	89.5	—	Pore engineering, metal doping	63
Macro-UiO-67-ILs	Lauric (dodecanoic) and myristic acid	FFA esterification	Methanol reflux, 65 °C, 30 min	99	Not reusable (framework damage on reflux)	Pore engineering, ligand functionalization	64
3AIL@NH_2_-UiO-66	Oleic acid; also, triglyceride transesterification	FFA esterification + transesterification	75 °C, 7 h, 15.3 : 1 MeOH : OA, 7.7 wt%	96.1 (esterification); >80 (transesterification)	6	Ligand functionalization, composite formations	65
[HSO_3_-BMIM][HSO_4_]@NH_2_-UiO-66	Butyric acid + glycerol	Model reaction	80 °C, 12 h	62.1	11 (67% of initial activity retained)	Ligand functionalization, composite formations	69
[(CH_2_COOH)_2_IM]HSO_4_@H-UiO-66	Oleic acid	FFA esterification	—	93.8	5	Ligand functionalization, metal defects	70
UiO-66(Zr)-NH_2_-green	Oleic acid	FFA esterification	60 °C	97.3	—	Ligand functionalization, metal defects	41
UiO-66(Zr)-NO_2_-green	Oleic acid	FFA esterification	60 °C	90.7	—	Ligand functionalization, metal defects	41
CF_3_SO_3_H-UiO-67-bpy	Isooctyl alcohol + acetic acid	Model reaction	90 °C, 18 h	98.6	5	Ligand functionalization	73
UiO-66/sulfamic acid	Oleic acid	FFA esterification	MeOH : OA 21.8 : 1, 7.6 wt%, 85 °C, 1.8 h	94.4 (yield); 95.5 (conversion)	5 (95.5 to 85.6%)	Ligand functionalization	74
UiO-66-(SO_3_H)_2_	Free fatty acids	FFA esterification	90 °C, 4 h, 15 : 1 MeOH : OA, 10 wt%; tolerates 10 wt% water	86.5	4 (−3.5%)	Ligand functionalization	75
UiO-66-NH_2_/ZnO/TiO_2_	Dairy waste scum oil	Transesterification	MeOH : oil 9.8 : 1, 2 wt%, 61 °C, 30 min, ultrasound	98.7	7	Composite formations	76
Fe_3_O_4_@UiO-66-NH_2_	Restaurant waste oil	Transesterification	Microwave, 8.8 min	98.2	7	Composite formations	87
H_3_PW_12_O_40_@UiO-67 (HPW@UiO-67)	Acetic acid + n-butanol (model esterification)	Model reaction	—	72.3	4	Ligand functionalization, composite formations	85
Ag_1_(NH_4_)_2_PW_12_O_40_/UiO-66	Lauric acid	FFA esterification	Lauric acid : MeOH 1 : 15, 10 wt%, 150 °C, 3 h	75.6	4	Composite formations	78
NiHSiW/UiO-66	Oleic acid	FFA esterification	—	86.7	8	Composite formations	84
Ce-BDC@HSiW@UiO-66	Oleic acid	FFA esterification	OA : MeOH 1 : 30, 0.2 g, 130 °C, 4 h	81.5	6	Composite formation, ligand functionalization	79
Ag@MOF-801	Triglycerides	Transesterification	10 wt%, 180 °C, 8 h	69	—	Composite formations	86
ROL@UiO-66-NH_2_	Waste oils	Transesterification	EtOH : oil 15 : 1	82	5	Enzyme immobilization	92
Zr-MOF/PVP	Castor oil	Transesterification	12 h	83	7	Enzyme immobilization	88

The studies surveyed here differ in how closely their test reaction resembles biodiesel synthesis, and this distinction is maintained throughout. Transesterification of a genuine triglyceride feedstock is the target transformation. Esterification of an isolated fatty acid such as oleic or lauric acid is also a real biodiesel reaction, since high-FFA feedstocks require it, but it does not test the diffusional constraint that governs triglyceride conversion, the substrate being roughly a third the molecular volume of a triglyceride and able to enter pores a triglyceride cannot. Non-lipid probe reactions benzaldehyde acetalization, levulinic acid conversion, short-chain ester synthesis interrogate the same Lewis and Brønsted acid sites and are useful for establishing structure activity relationships at the node and linker, but their substrates are smaller again and they carry no information about mass transfer, which this review identifies as the dominant constraint on catalyst design. Results from such reactions are identified as model reactions wherever they appear, are used only to support mechanistic argument, and are never reported as biodiesel performance; and the reaction type of every system compiled in Table 1 is given there. Applying this distinction across the literature is itself informative: of the systems listed in Table 1, only about a third test genuine triglyceride transesterification, half use isolated fatty acids, and the remainder use non-lipid probe reactions.

### 2.1 The pristine framework as a catalytic baseline

The first zirconium-based MOF, UiO-66, was reported by Cavka *et al.* and named after the University of Oslo, where it was first synthesized.^38^ It is constructed from Zr_6_O_4_(OH)_4_ secondary building units, each connected to twelve benzene-1,4-dicarboxylate (BDC) linkers one of the highest coordination numbers found in any MOF, and the origin of the framework's exceptional stability. The UiO series withstands most organic solvents and remains crystalline after exposure to external pressures of ∼1 GPa (10 tons cm^−2^), making it well suited to the demanding conditions of biodiesel synthesis.^38^ This architecture invited isoreticular expansion: replacing BDC with progressively longer dicarboxylate linkers yields UiO-67 and UiO-68, increasing the pore aperture from ∼6 Å in UiO-66 to ∼8 Å and ∼10 Å, and the BET surface area from ∼1167 m^2^ g^−1^ to ∼3000 and ∼4170 m^2^ g^−1^, respectively. The UiO series withstands most organic solvents and mechanical pressures approaching 1 GPa, making it well suited to the demanding conditions of biodiesel synthesis.^39,40^

This structural robustness, however, comes at a catalytic cost. In an ideal, defect-free framework each Zr_6_ node is coordinatively saturated by twelve carboxylate linkers, every Zr^4+^ center being eight-coordinate through four carboxylate oxygens and four µ_3_–O/µ_3_–OH bridges, so no vacancy is available to bind a substrate and the material is essentially inactive for both esterification and transesterification.^38,40^ The pristine framework therefore serves in this review as a structural and catalytic baseline rather than as a modification strategy in its own right: it defines the porosity, stability and node chemistry that the five strategies discussed below seek to modify. Each of those strategies can be understood as a different route to generating or exploiting catalytically accessible sites within this architecture by removing or substituting components of the node, by enlarging the pore network, by functionalizing the linker, by combining the framework with a second phase, or by creating an enzyme within it. In practice, conventionally synthesized UiO-66 is never defect-free: Abou-Elyazed *et al.* measured 40% oleic acid conversion over solvothermal prepared UiO-66(Zr) under conditions identical to those used for its modified analogues, a weak but non-zero activity attributable to adventitious missing-linker defects.^41^

### 2.2 Metal defect and node modification

Catalytic activity in Zr-MOFs originates in departures from the ideal structure. Missing-linker and missing-cluster defects generate coordinatively unsaturated Zr^4+^ centres that act as Lewis acid sites, together with terminal –OH groups that act as Brønsted sites, and it is these defect-derived sites that allow UiO-66 and its derivatives to catalyse both the esterification of free fatty acids and the transesterification of triglycerides, with conversions exceeding 90% and good reusability over several cycles.^32,42,43^ Any deviation from the ideal crystallographic structure missing metal nodes, missing linkers, or incomplete Zr^4+^ coordination is termed a defect in Zr-MOFs.^44^ Coordinatively unsaturated sites (CUS), which serve as Lewis acid centers for transesterification, are created when such defects are deliberately introduced. In addition to increasing pore apertures and exposing unsaturated Zr^4+^ ions, missing linkers significantly improve accessibility for bulky triglycerides.^45,46^ A distinction should be drawn here between two routes to the same kind of site. In UiO-66 the Zr_6_ node is 12-connected, so coordinatively unsaturated Zr^4+^ appears only where linkers are absent that is, only through genuine defects. In NU-1000 and MOF-808 the node is 8- and 6-connected by design, the remaining coordination positions carrying terminal OH and OH_2_ groups together with modulator carboxylates that are displaced on activation; these frameworks are therefore not defective UiO-66 but distinct topologies in which open Zr^4+^ sites and Brønsted acidic node protons are intrinsic.^47^ Both routes expose comparable sites, but the lower connectivity of MOF-808 and NU-1000 couples a higher density of accessible Zr^4+^ to substantially wider pores than 12-connected UiO-66, which is the usual explanation for their greater activity in acid-catalysed reactions.^48^ Because of the increased pore space and acid site density, these defects provide better catalytic activity than 12-connected UiO-66.

**Fig. 2:**
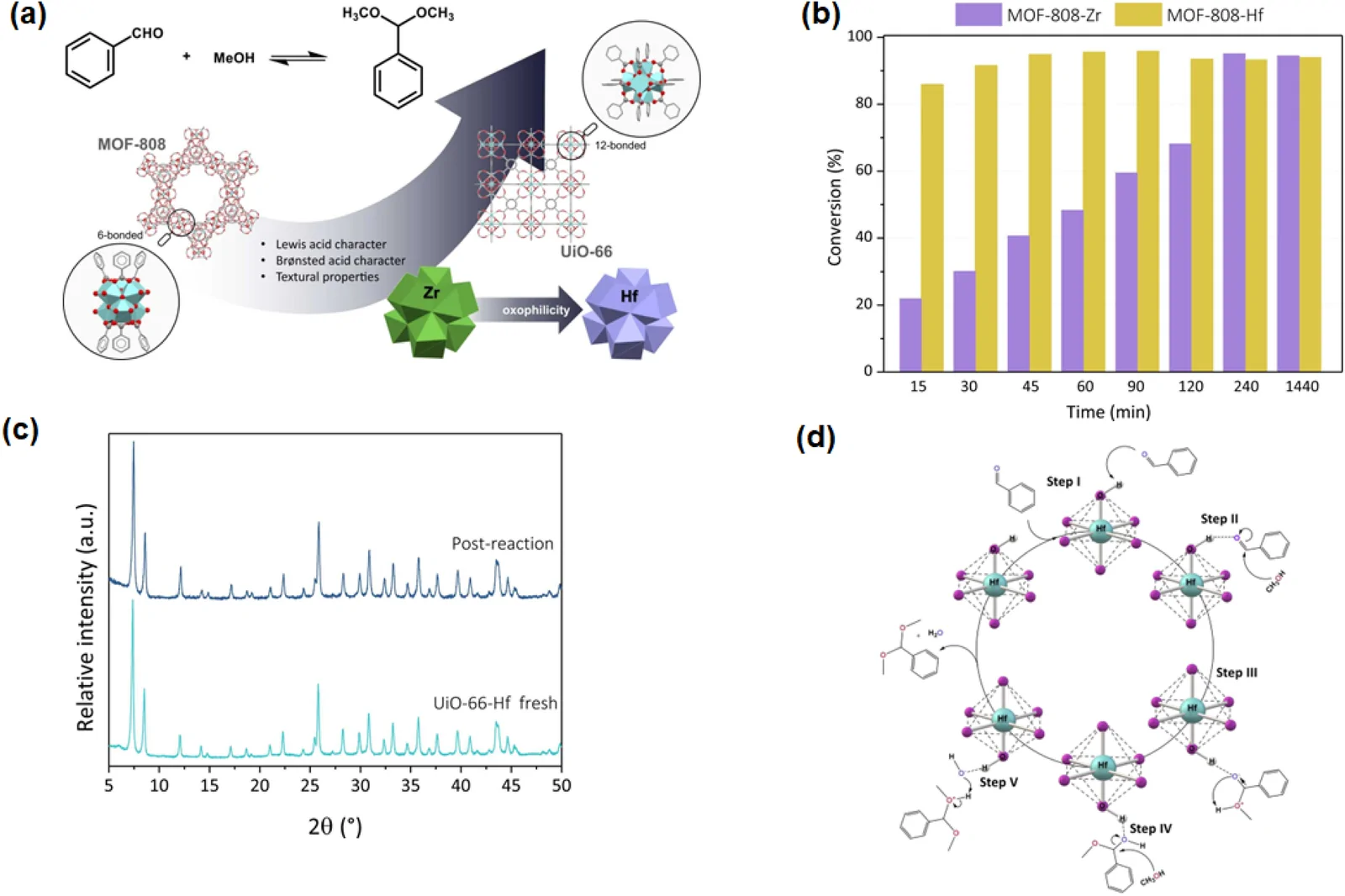
Effects of metal node substitution for acetalization in Zr/Hf-MOFs. (a) MOF-808 *vs.* UiO-66 structural schematic with 6-connected and 12-connected Zr_6_ clusters, along with a comparison of the oxophilicity and acid character of Zr and Hf nodes. (b) Benzaldehyde conversion *vs.* time for MOF-808-Zr and MOF-808-Hf, showing faster kinetics for Hf-MOF. (c) XRD patterns of UiO-66-Hf before and after reaction confirming structural stability. (d) Proposed 5-step mechanism for acetalization over Hf-MOF active sites.^49^ This figure has been adapted/reproduced from ref. 49 with permission from Elsevier, copyright 2024.

Another tactic is to substitute metal nodes. Zr- and Hf-based UiO-66 and MOF-808 were compared by García-Rojas *et al.* for benzaldehyde acetalization.^49^ Zr-MOFs and Hf-MOFs were both produced solvothermal, as seen in Fig. 2a. As shown in Fig. 2b, the difference between the two metals is kinetic rather than thermodynamic: MOF-808-Hf reached 85% benzaldehyde conversion in 15 min against 21% for MOF-808-Zr under the same conditions (50 wt% catalyst, benzaldehyde 1 mmol, methanol 75 mmol, 30 °C), corresponding to productivities of 22 and 4 mmol g^−1^ h^−1^, although after 24 h both materials converged to similar conversions. This rate enhancement is attributed to the greater oxophilicity of Hf^4+^ relative to Zr^4+^ the Hf–O bond dissociation enthalpy is 802 kJ mol^−1^ against 776 kJ mol^−1^ for Zr–O which confers stronger Brønsted acid character on the node and accelerates substrate activation. Consistent with this, comparative Brønsted acidity measurements across the UiO-66(Zr, Ce, Hf) series confirm that the Hf analogue possesses the strongest node acidity reaching a turnover frequency ninety times that of the Zr framework in glycerol acetalization providing an independent basis for the superior activity of Hf-MOFs in acid-catalyzed transformations.^50^ UiO-66-Hf also proved the more robust catalyst, sustaining activity over five cycles compared with two for MOF-808-Hf, and post-reaction XRD (Fig. 2c) confirmed retention of crystallinity with no detectable Hf leaching. To form the acetal product, the five-step mechanism proposed in Fig. 2d involves protonation of benzaldehyde by Hf–OH, nucleophilic attack by methanol, and elimination of water. Although acetalization is a model reaction rather than a biodiesel transformation, it probes the same Lewis and Brønsted acid sites responsible for transesterification; the defect and metal-substitution principles established here are therefore expected to transfer to triglyceride conversion, and the enhanced oxophilicity of Hf^4+^ over Zr^4+^ provides a rational design handle for acid-site strengthening in Zr-MOF biodiesel catalysts.^49^

**Fig. 3:**
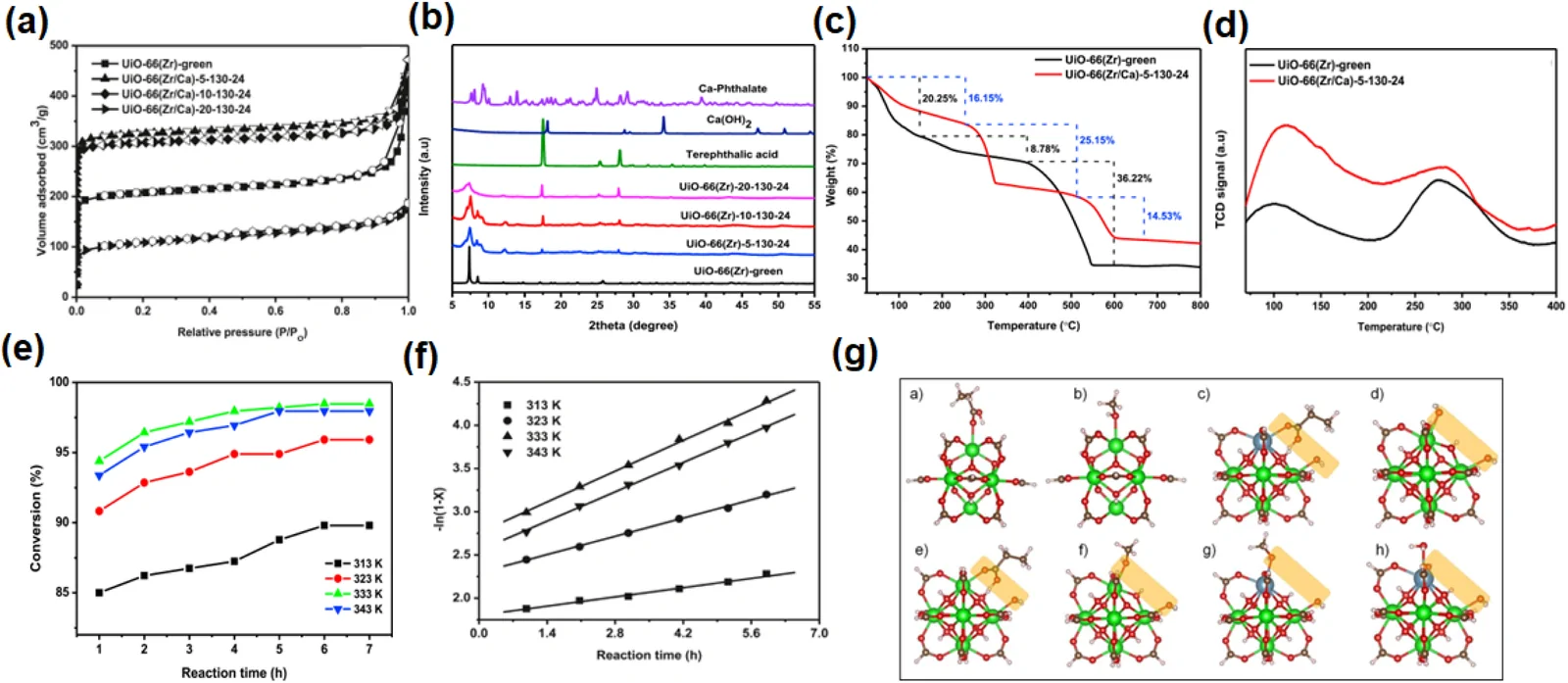
Characterization and catalytic performance of 5% Ca^2+^-doped UiO-66(Zr). (a) N_2_ adsorption–desorption isotherms showing BET surface area of 1120 m^2^ g^−1^. (b) XRD patterns confirming crystallinity and successful Ca^2+^ incorporation. (c) TGA curves showing >100 °C improvement in thermal stability for Ca^2+^-doped UiO-66. (d) NH_3_-TPD profiles indicating increased acid site density. (e) Oleic acid conversion *vs.* time at 313–343 K. (f) Plots of −ln(1 − *x*) *vs.* time for activation energy calculation, *E*_a_ ≈ 37 kJ mol^−1^. (g) DFT-optimized structures showing increased metal–oxygen bond length and favorable defect formation upon Ca^2+^ doping.^43^ This figure has been adapted/reproduced from ref. 43 with permission from Elsevier, copyright 2022.

The crucial significance of linker defects in catalytic performance was confirmed by thermogravimetric analysis (TGA) of UiO-66 and NH_2_-UiO-66, which showed that increasing the number of missing linkers increased rate constants for levulinic acid esterification.^51^ Defected Zr-MOFs showed almost nine times more activity in the same reaction, according to Cirujano *et al.*^52^ Activity was highly dependent on defect density and framework dimensionality. Another method of defect engineering is metal doping. 5% Ca^2+^-doped UiO-66(Zr) was created by Abou-Elyazed *et al.* using a solvent-free technique at 130 °C for 24 hours.^43^ The catalyst had a high BET surface area of 1120 m^2^ g^−1^, as seen in Fig. 3a. Ca^2+^ incorporation without loss of crystallinity was established by XRD and XPS (Fig. 3b). Catalytically, the important result is the TPD data (Fig. 3d): doping raises acid and base site densities together, so that methanol and the carbonyl group of oleic acid are activated at neighbouring sites. Pristine UiO-66 does not have this bifunctionality, and it explains the low activation energy of ∼37 kJ mol^−1^. For both the first and second stages of weight reduction, TGA in Fig. 3c demonstrated an improvement in thermal stability of >100 °C. While DFT calculations in Fig. 3g suggested longer Zr–O bonds and favorable defect development, kinetic studies in Fig. 3e and f yielded an activation energy of ∼37 kJ mol^−1^. Defect engineering can lead to high-performance biodiesel catalysts, as demonstrated by the Ca^2+^-doped catalyst's 98% biodiesel output at 333 K.

### 2.3 Pore engineering

Since triglycerides and free fatty acids are large molecules that need mesopores for effective mass transfer, pore size and accessibility are crucial for the production of biodiesel.^53,54^ Pore engineering for Zr-MOFs is usually accomplished by sulfonation, defect formation, modulator-induced mesoporosity, and linker extension.^47,55–59^ Brønsted acidity can also be raised by anchoring sulfonic acids to the framework: *p*-toluenesulfonic acid supported on UiO-66 converted 97.1% of palmitic acid and 95.8% of oleic acid at 100 °C (8 : 1 methanol : acid, 4 h), against only 25.2% for unmodified UiO-66 under identical conditions, confirming that the sulfonic acid rather than the node supplies most of the activity.^60^

**Fig. 4:**
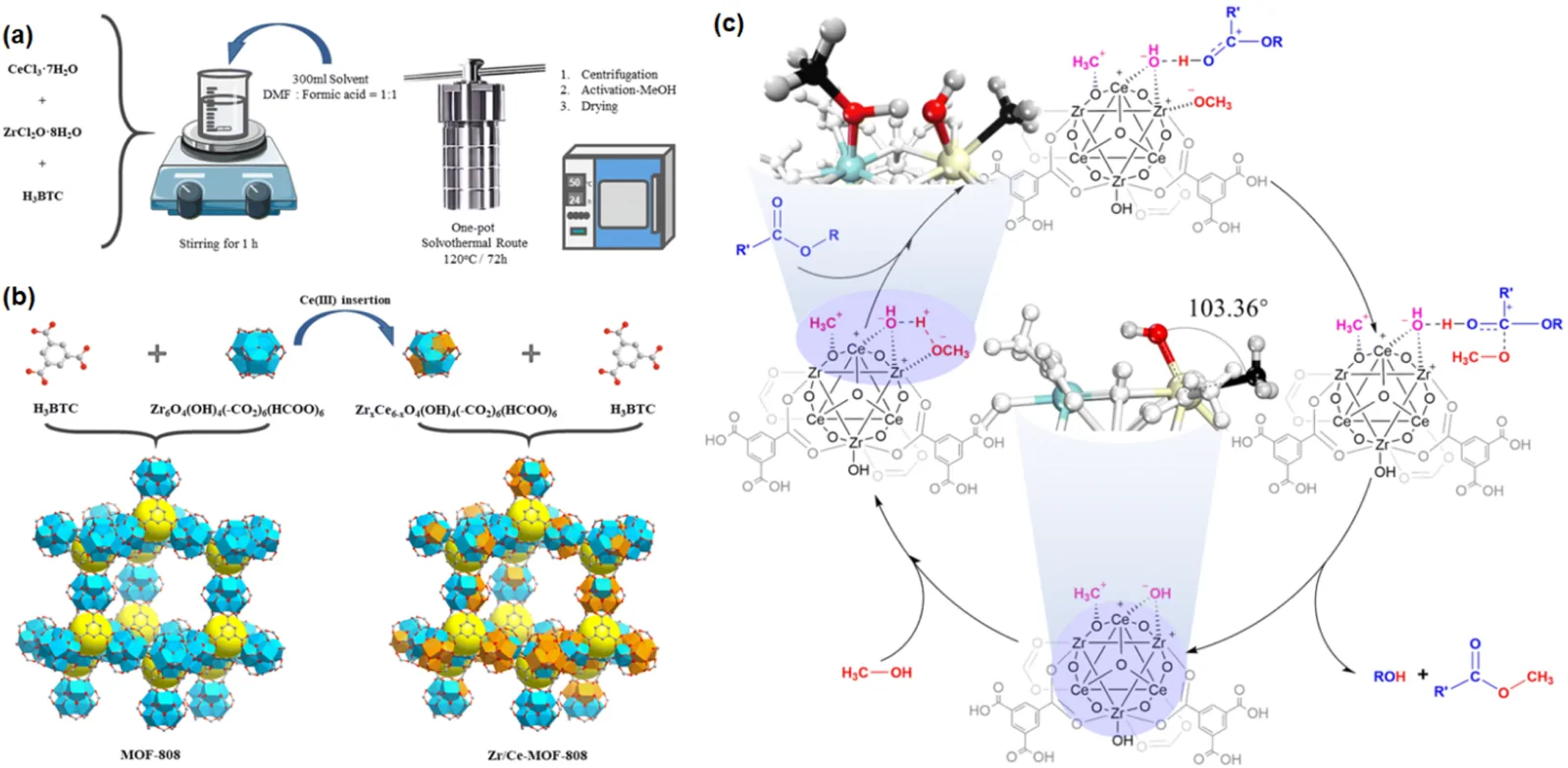
Synthesis and mechanism of Zr/Ce-MOF-808 bimetallic catalysts. (a) One-pot solvothermal synthesis route of Zr/Ce-MOF-808 using CeCl_3_·7H_2_O, ZrOCl_2_·8H_2_O and H_3_BTC in DMF/formic acid at 120 °C for 72 h. (b) Structural comparison of pristine MOF-808 and Ce^3+^-inserted Zr/Ce-MOF-808 showing bimetallic cluster formation. (c) Proposed mechanism for transesterification on Zr–OH–Ce bridging sites, where the hydroxyl group acts as a nucleophile to activate methanol and attack the carbonyl of triglycerides.^63^ This figure has been adapted/reproduced from ref. 63 with permission from the Royal Society of Chemistry, copyright 2024.

The 6-connected Zr_6_ clusters of MOF-808, bearing terminal µ_3_–OH, Zr–OH_2_, and Zr–OH groups that function as Brønsted acid sites, make this framework particularly appealing.^56,61^ However, the micropores of pristine MOF-808 are too narrow to admit microalgal lipid molecules, which are themselves roughly 1.8 nm across, and the Zr sites in its saturated secondary building units are too weakly acidic.^47^ To overcome both limitations, Qian *et al.* exploited ligand competition: by modulating the basicity of DMF with formic acid, the coordination of benzene-1,3,5-tricarboxylic acid to the Zr clusters was perturbed, generating three distinct types of functional defect.^55^ This expanded the pore size from 1.63 nm to 5.34 nm and increased catalyst acidity by ∼23% while preserving high porosity. Density functional theory identified the active catalytic sites as the exposed Zr^4+^ defect centers. The optimal catalyst, MOF-808-3/1, reached 96.2% FAME yield from microalgal lipids at 200 °C (20 : 1 methanol : oil, 2 wt% catalyst, 60 min), but the more consequential result is that its conversion exceeded 90% across the entire 100–200 °C range, giving 90.4% at 100 °C where the non-hierarchical MOF-808-1/1 managed only 68.1% under identical conditions. Operating at 100 °C rather than 200 °C lowers the saturated vapor pressure of methanol from 4.0 to 0.35 MPa, an order-of-magnitude reduction in reactor pressure, and the catalyst lost only 2.5% of its conversion over six cycles. Retention of crystallinity, with increased intensity at 2*θ* = 4.4°, was confirmed by XRD. The proposed mechanism involves adsorption of methanol on the Zr sites to form methoxy intermediates, which then attack the triglyceride carbonyl nucleophilically to yield FAME and diglycerides.^62^ Bimetallic doping improves pore characteristics even further. Zr/Ce-MOF-808 was created by Qian *et al.* using solvothermal synthesis to integrate Ce^3+^ into MOF-808.^63^ As seen in Fig. 4a and b, Zr/Ce (1 : 1) had more bridging –OH groups (Fig. 4c) and a larger BET surface area of 1912 m^2^ g^−1^ compared to 1125 m^2^ g^−1^ for pristine MOF-808. The bimetallic cluster structure and one-pot synthesis of Zr/Ce-MOF-808 are shown in Fig. 4a and b. The Zr–OH–Ce bridging hydroxyl functions as a nucleophilic site, absorbing electron density from both metals to activate methanol and attain 89.5% conversion at 100 °C and 0.35 MPa, as illustrated in Fig. 4c.^63^

**Fig. 5:**
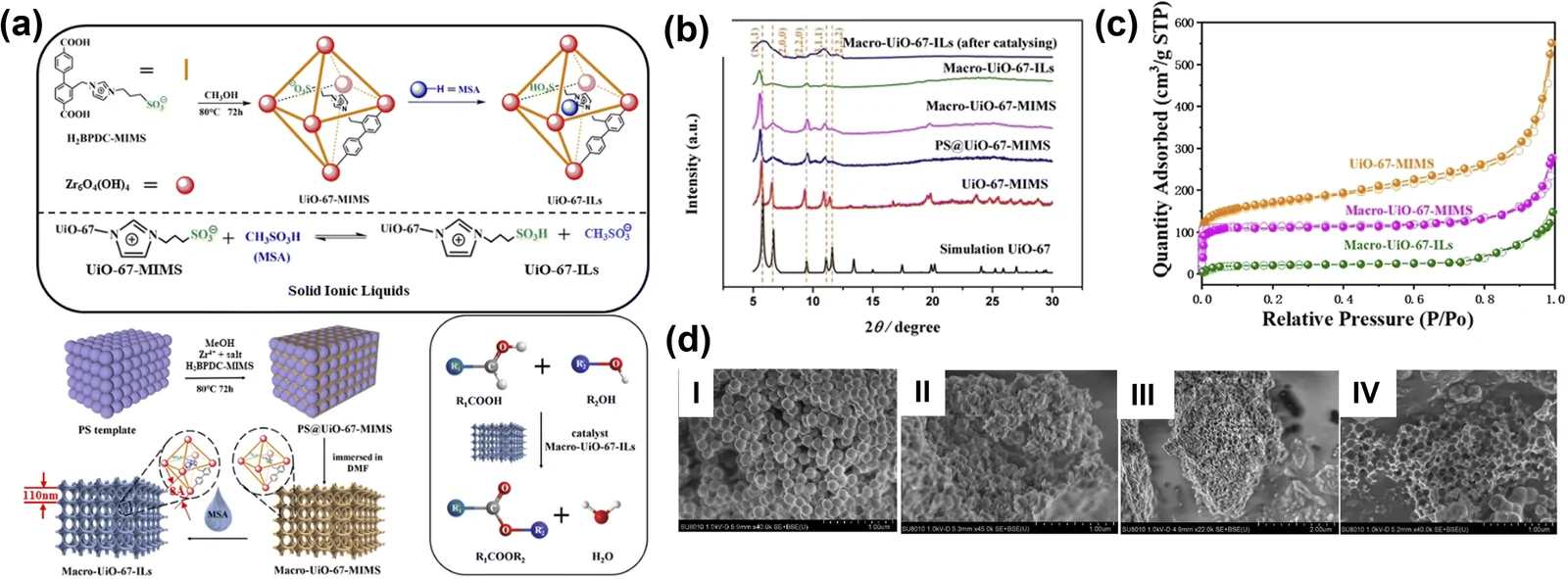
(a) Synthesis of UiO-67-MIMS and UiO-67-ILs by post-modification of UiO-67 with imidazolium methanesulfonate MIMS and ionic liquid HLa. (b) XRD patterns confirming crystallinity after catalysis. (c) N_2_ adsorption–desorption isotherms showing meso/macro porosity. (d) SEM images of: (I) UiO-67-MIMS, (II) macro-UiO-67-MIMS, (III) PS@UiO-67-MIMS, (IV) macro-UiO-67-ILs.^64^ This figure has been adapted/reproduced from ref. 64 with permission from the Royal Society of Chemistry, copyright 2023.

Micropores for active sites and macropores for the mass transport of large triglycerides are combined in hierarchical pore engineering. Following template removal, 1–110 nm pores were created using reticular chemistry with a polystyrene template, as illustrated in Fig. 5a. Li *et al.* covalently grafted sulfonated imidazolium ionic liquids onto UiO-67 using a large H_2_BPDC-imidazolium methanesulfonate (MIMS) linker. The template approach created hierarchical micro/mesopores while maintaining UiO-67 crystallinity, as seen in Fig. 5a and b. This improved accessibility of active Brønsted acid sites and increased conversion of bulky fatty acids to FAME.^64^ The UiO-67 structure with its distinctive (111) and (200) peaks was retained, according to PXRD in Fig. 5b, although PS inclusion caused the intensity to drop. While Macro-UiO-67-MIMS displayed hierarchical micro/mesopores, microporous UiO-67-MIMS retained ∼1.2 nm pores, according to BET analysis in Fig. 5c. The macroporous network created by template removal was shown by SEM in Fig. 5d. Macro-UiO-67-ILs, bearing zwitterionic MIMS–MSA ionic liquid sites, gave 99% esterification yield in 30 min under methanol reflux at 65 °C. The comparison that isolates the pore effect is with its own non-hierarchical counterpart, which carries essentially the same components but retains only micropores: this gave 64% for dodecanoic acid and 58% for myristic acid. Bulk methanesulfonic acid reached 91% and 88% on the same substrates while using seven times more acid (30 mmol against 4.35 mmol buffered in the framework), and the uncatalysed reaction gave only ∼22% after 24 h. Macropores therefore lower diffusion resistance and the acidic IL sites supply reactivity, but the benefit does not extend to durability: the authors report widened PXRD reflections and reduced activity after reaction, attributing framework damage to the heating reflux and concluding that the catalyst may not be reusable. The findings verify that pore expansion in Zr-MOFs is essential for producing biodiesel from large feedstock effectively.

### 2.4 Linker/ligand functionalization

Without changing the metal cluster, linker functionalisation adds active groups to the organic ligand of Zr-MOFs. On benzene dicarboxylic acid linkers, common substituents include –NH_2_, –NO_2_, and –OH, which enhance interaction with polar reactants and offer anchoring sites for acidic species.^65^

**Fig. 6:**
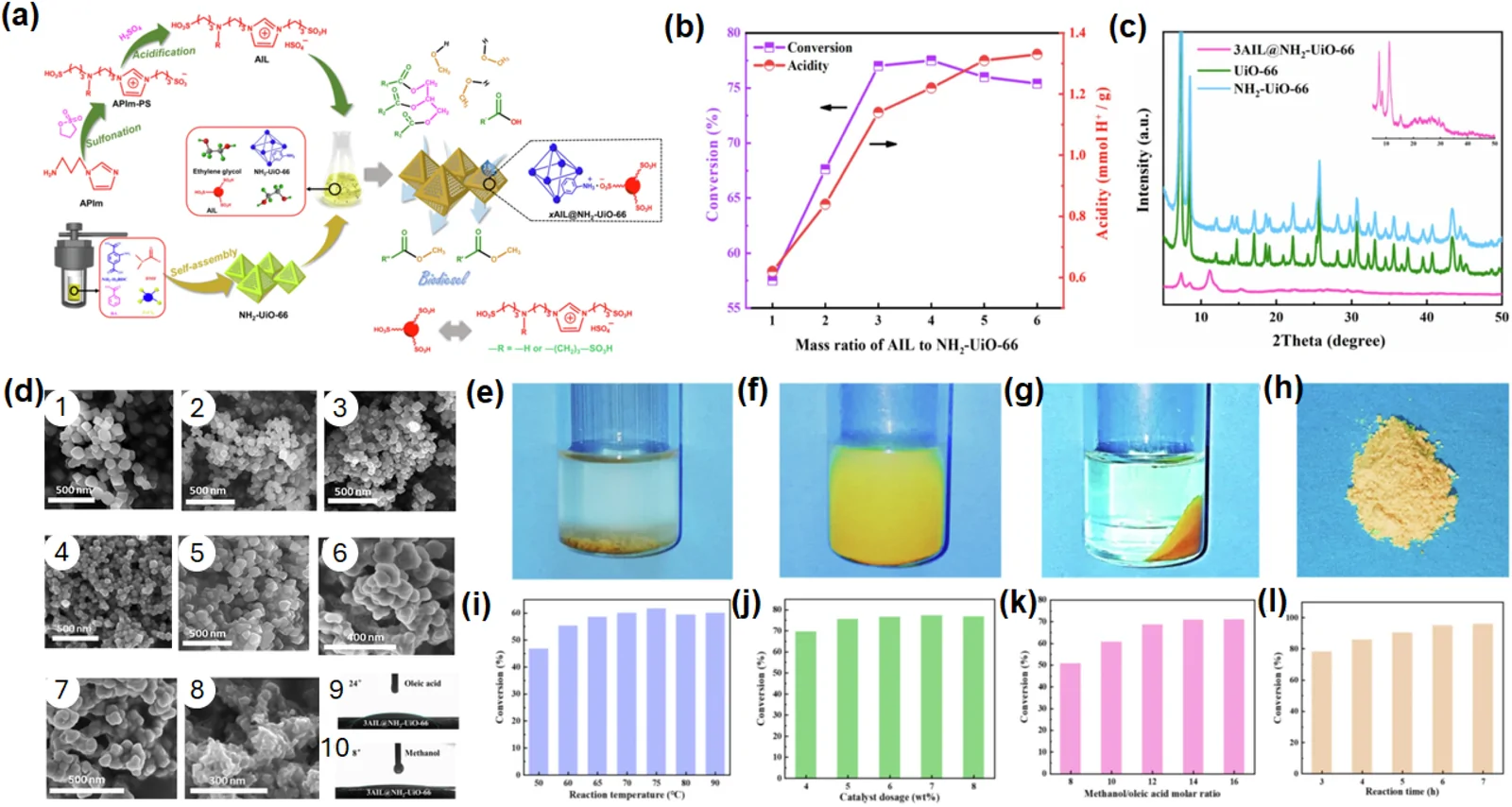
Synthesis and characterization of AIL@NH_2_-UiO-66 for biodiesel production. (a) Schematic of AIL preparation from APIm and PS, followed by grafting onto NH_2_-UiO-66. (b) Effect of AIL mass ratio on acidity and oleic acid conversion: acidity increases monotonically across *x* = 1–6 while conversion peaks at *x* = 3–4. (c) PXRD patterns of UiO-66, NH_2_-UiO-66, and 3AIL@NH_2_-UiO-66 showing retained crystallinity. (d) SEM images of (1) UiO-66, (2) NH_2_-UiO-66 and (3–8) xAIL@NH_2_-UiO-66 for *x* = 1–6, showing retention of the regular octahedral morphology up to *x* = 3 and progressive surface roughening and loss of morphology at higher loading; (9,10) contact angles of oleic acid (24°) and methanol (5°) on 3AIL@NH_2_-UiO-66. (e–h) Photographs of reaction mixture at start, during reaction, after completion, and regenerated catalyst. (i–l) Single-factor of optimization of reaction temperature, catalyst dosage, methanol : oleic acid molar ratio, and reaction time for 3AIL@NH_2_-UiO-66.^65^ This figure has been adapted/reproduced from ref. 65 with permission from Elsevier, copyright 2022.

Although they have significant viscosity and separation problems, acidic ionic liquids (AILs) are potential acid catalysts.^66–68^ By grafting sulfonic acid-functionalized AILs onto NH_2_-UiO-66 *via* acid–base coordination between –SO_3_H and –NH_2_ groups, Lu *et al.* were able to overcome this, in which 1-(3-aminopropyl)imidazole and 1,3-propanesulfonate were used to create the AIL, which was subsequently protonated with H_2_SO_4_.^65^. NH_2_-UiO-66 was made solvothermal from ZrCl_4_ and NH_2_-H_2_BDC, and *x*AIL@NH_2_-UiO-66 was obtained by treating it with ethylene glycol (Fig. 6a). As shown in Fig. 6b, acidity rises monotonically with AIL loading across the whole series (6AIL > 5AIL > 4AIL > 3AIL > 2AIL > 1AIL), but conversion does not: it tracks acidity only up to *x* = 4 and then falls, giving the order 4AIL ≈ 3AIL > 5AIL > 6AIL > 2AIL > 1AIL. XRD shows why samples with *x* ≥ 4 depart from the NH_2_-UiO-66 pattern, the altered framework constricting the pore network and limiting contact between the acid sites and the reactants. This system is therefore a direct demonstration of the acid-density/accessibility trade-off: beyond a certain loading, adding acid sites removes access to them faster than it adds activity (Fig. 6c and d). Single-factor optimisation (Fig. 6i–l) gave 75 °C, a 14 : 1 methanol : oleic acid molar ratio, 5 wt% catalyst and 6 h, at which oleic acid conversion reached 95.2%; response surface modelling raised this to an experimental 96.1% against a predicted 98.0% (7.67 wt%, 15.29 : 1, 7 h, 75 °C). The same catalyst gave above 80% biodiesel yield in transesterification. Reusability was good rather than complete: oleic acid conversion fell from 96.9% to 90.4% over six runs, so modest ligand functionalization balances acidity against structural stability without eliminating deactivation.

UiO-66 with more defects and adjustable acidity is produced by solvent-free mechanochemical synthesis. To create UiO-66-green, UiO-66-NH_2_-green, and UiO-66-NO_2_-green, Abou-Elyazed *et al.* ground ZrOCl_2_·8H_2_O with BDC, NH_2_-BDC, or NO_2_-BDC and heated the mixture at 130–150 °C.^41^ NH_3_-TPD/CO_2_-TPD showed that –NH_2_ donates electrons to Zr through the benzene ring, decreasing Zr^4+^ Lewis acidity while increasing the Lewis basicity of Zr–OH/Zr–O–Zr and the Brønsted basicity of –NH_2_; the –NO_2_ group has the opposite effect and gives the highest acid strength. For oleic acid esterification at 60 °C, conversions were 86.3% for UiO-66-green, 90.7% for UiO-66-NO_2_-green, and 97.3% for UiO-66-NH_2_-green. The superior activity of the –NH_2_ variant arises from dual activation: the –NH_2_ Brønsted base activates methanol while the Zr^4+^ Lewis acid activates the carbonyl of oleic acid. The green-synthesized frameworks also displayed a wider surface area and improved pore distribution, with an activation energy of 15 kJ mol^−1^ and an endothermic reaction profile.^41^

Immobilizing acidic ionic liquids (AILs) on NH_2_-UiO-66 combines the stability of the MOF with the Brønsted acidity of the IL. Zheng *et al.* anchored [HSO_3_-BMIM][HSO_4_] onto NH_2_-UiO-66 through an acid–base reaction and applied the resulting material to the esterification of butyric acid with glycerol—a model esterification rather than a transesterification to FAME. Anchoring to the MOF suppressed IL leaching and giving 62.2% butyric acid conversion under optimized conditions (80 °C, 12 h) and retention of 67.4% of the initial esterification activity after eleven consecutive cycles. AIL grafting therefore extends operational life over an unusually large number of runs, but does not prevent a substantial loss of activity across them.^69^

A complementary approach exploits ligand defects in hierarchically porous H-UiO-66. Ye *et al.* introduced the dicarboxyl-functionalized imidazolium ionic liquid [(CH_2_COOH)_2_IM]HSO_4_ into the framework by approximate ligand substitution, in which one –COO^−^ group of the IL coordinates in bidentate fashion to unsaturated Zr^4+^ defect sites, yielding [(CH_2_COOH)_2_IM]HSO_4_@H-UiO-66. The catalyst is bifunctional: Brønsted acidity is supplied by the HSO_4_^−^ anion of the IL and Lewis acidity by the defect Zr^4+^ centers, so that the carbonyl of oleic acid is activated by the Zr node while methanol is activated by the Brønsted site. Esterification of oleic acid with methanol gave a conversion of 93.8%, with activity retained at 90.9% after five cycles.^70^

**Fig. 7:**
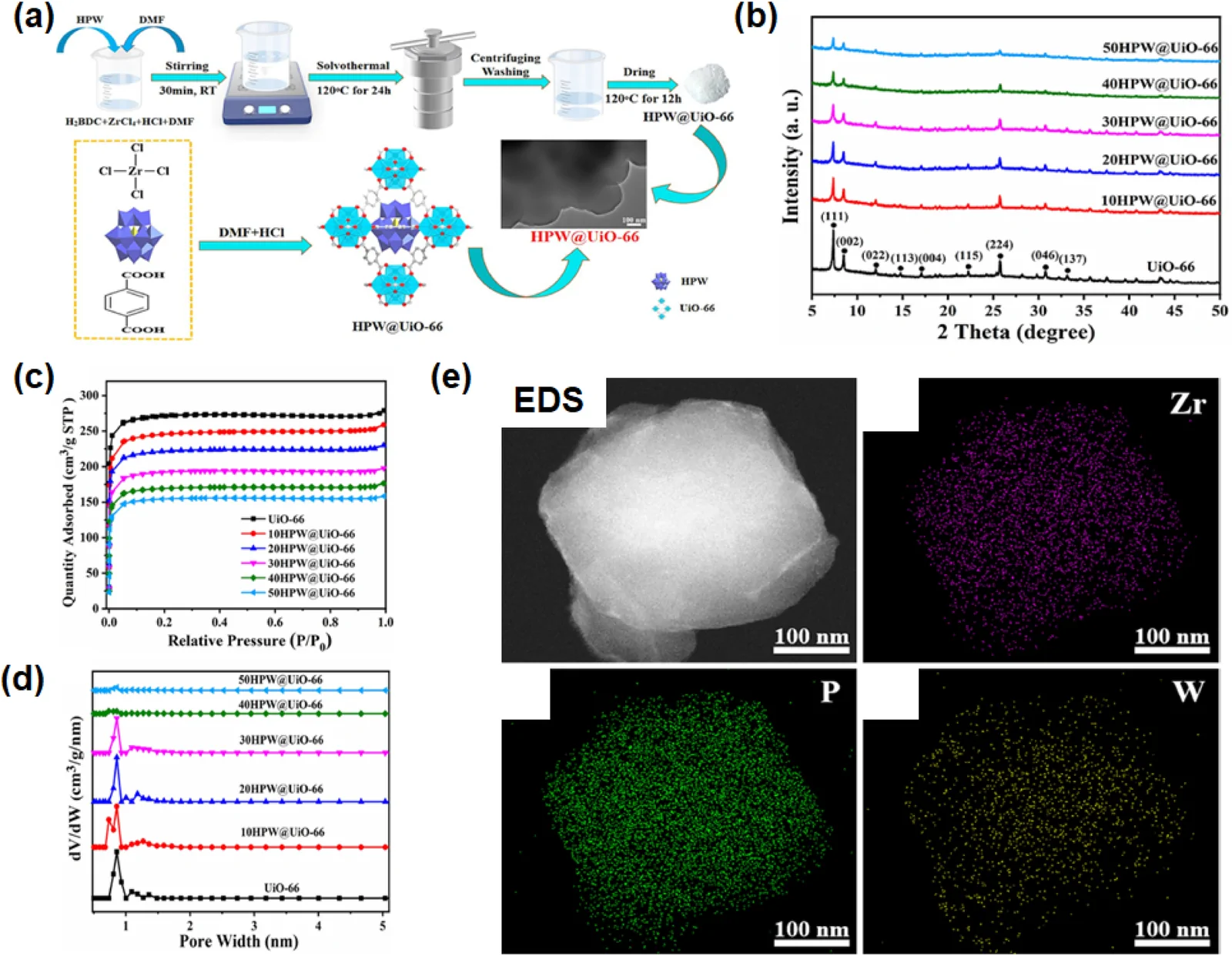
Synthesis and characterization of HPW@UiO-66 composites. (a) Schematic of one-pot hydrothermal synthesis showing encapsulation of HPW in UiO-66 cages. (b) PXRD patterns of UiO-66 and HPW@UiO-66 with 10–50 wt% loading, showing decreasing intensity but retention of framework and absence of free HPW peaks. (c) N_2_ adsorption–desorption isotherms and (d) pore size distribution curves showing microporous structure with decreasing surface area at higher HPW loading. (e) TEM image of 30HPW@UiO-66 with EDS elemental mapping of Zr, P, and W confirming uniform dispersion of HPW within MOF matrix.^72^ This figure has been adapted/reproduced from ref. 72 with permission from Elsevier, copyright 2022.

Grafting heteropolyacids onto the amine linker avoids both leaching and pore obstruction. Mulik and Bokade reacted phosphotungstic acid (HPW) with UiO-66-NH_2_ to give UiO-66-NH_2_-HPW, in which ATR-FTIR and XPS confirmed chemical bonding between the –NH_2_ group and the HPW cluster. NH_3_-TPD showed markedly greater acidity than pristine UiO-66-NH_2_ (total acidity 0.436 against 0.28 mmol g^−1^), and although grafting reduced the surface area from 907 to 669 m^2^ g^−1^ and the pore diameter from 3.79 to 3.39 nm, sufficient accessibility was retained for the framework to remain active. In ethanol at 150 °C with 20 wt% catalyst, furfuryl alcohol conversion reached 97 mol% after 4 h, though the useful products accounted for only part of this 31 mol% ethyl furfuryl ether and 33 mol% ethyl levulinate, the balance being lost largely to humins. This is a model reaction rather than a biodiesel transformation, and it is cited here only to show that heteropolyacid grafting to the amine linker preserves active-site accessibility.^71^ In a complementary approach, Ma *et al.* varied the HPW loading in UiO-66 from 10 to 50 wt%. PXRD (Fig. 7b) showed progressively weaker UiO-66 reflections with no free HPW peaks, confirming encapsulation rather than surface deposition, while N_2_ adsorption (Fig. 7c) gave a microporous surface area of 924 m^2^ g^−1^. The pore size distribution (Fig. 7d) is consistent with one HPW cluster per octahedral cage, giving pores of 0.7–1.2 nm that physically inhibit leaching, and TEM-EDS mapping (Fig. 7e) confirmed uniform dispersion of Zr, P, and W. 30HPW@UiO-66 achieved 80.2% conversion in the esterification of acetic acid with *n*-butanol over four stable cycles, a model esterification rather than a biodiesel transformation, but one that probes the same Brønsted acid sites.^72^

**Fig. 8:**
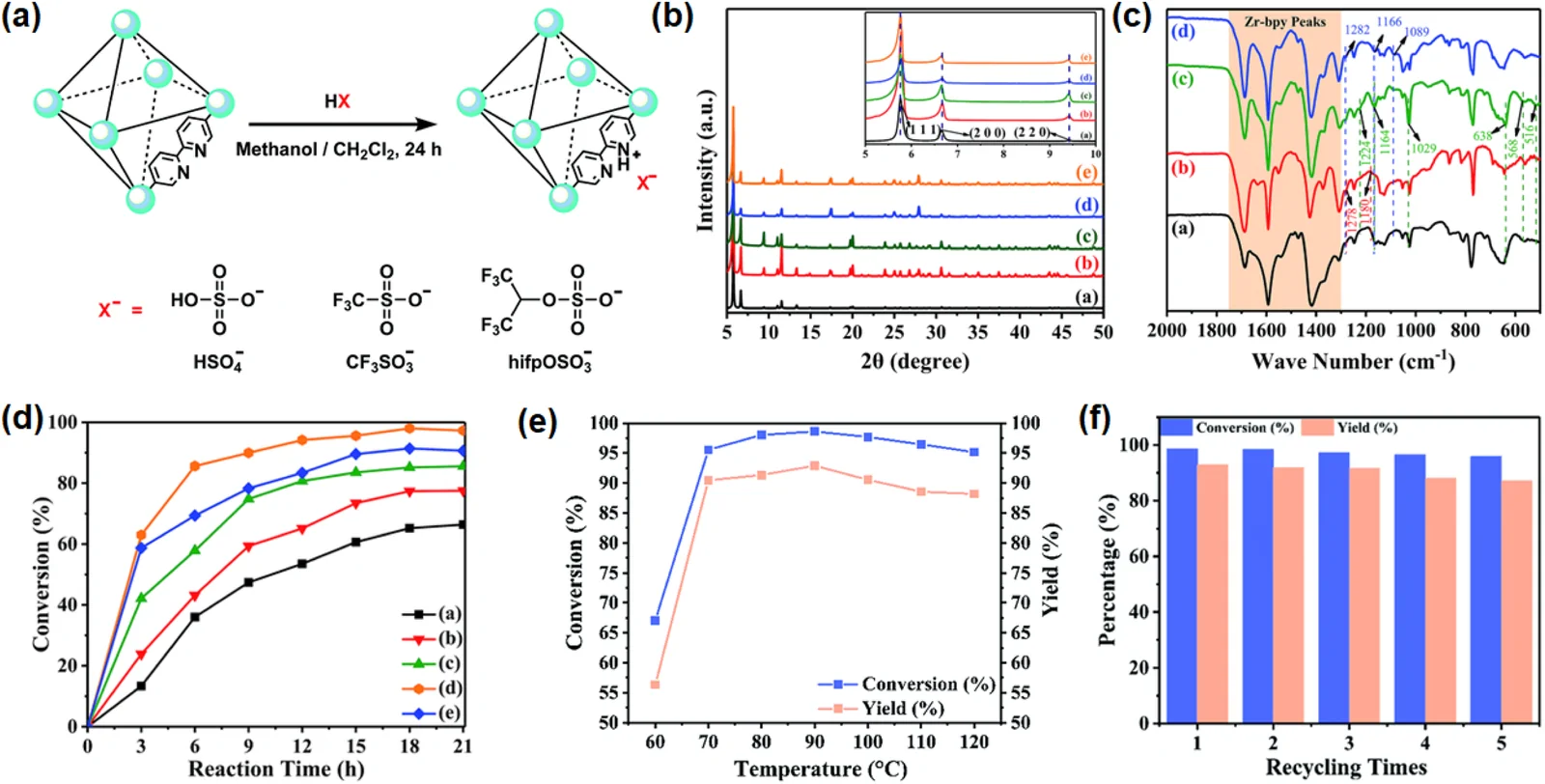
Acid-functionalized UiO-67-bpy catalysts for esterification of isooctyl alcohol with acetic acid. (a) Schematic of Brønsted acid grafting onto bipyridine N-sites with HSO_4_^−^, CF_3_SO_3_^−^, and hifpOSO_3_^−^ anions. (b) PXRD patterns of UiO-67-bpy and acid-functionalized variants showing retained crystallinity. (c) FTIR spectra confirming O=S=O, C–F, and C–S vibrations from grafted acids. (d) Conversion *vs.* reaction time for all catalysts, with CF_3_SO_3_H-UiO-67-bpy giving highest activity. (e) Effect of temperature on conversion and yield for CF_3_SO_3_H-UiO-67-bpy, with optimum at 90 °C. (f) Reusability test of CF_3_SO_3_H-UiO-67-bpy over 5 cycles showing stable conversion and yield.^73^ This figure has been reproduced from ref. 73 under a Creative Commons CC-BY license, copyright 2018 the authors, published by the Royal Society of Chemistry.

Brønsted acidity can also be achieved by post-synthetic alteration of N-containing linkers. To create acid-functionalized catalysts for esterification of isooctyl alcohol with acetic acid, Xu *et al.* protonated UiO-67-bpy using H_2_SO_4_, CF_3_SO_3_H, and hifpOSO_3_H.^73^ Without causing any harm to the framework, the bipyridine nitrogen sites coupled with Brønsted acids to form stable acid–base pairs (Fig. 8a). While FTIR in Fig. 8c revealed O=S=O, C–F, and C–S vibrations verifying acid grafting, PXRD in Fig. 8b verified crystallinity was maintained. CF_3_SO_3_H-UiO-67-bpy reached 98.6% conversion in 18 hours, according to kinetic studies in Fig. 8d, which was faster than HSO_4_^−^ and hifpOSO_3_^−^ versions. Maximum yield was obtained at 90 °C through temperature optimisation in Fig. 8e, and reusability experiments in Fig. 8f verified no leaching with consistent performance over five cycles.^73^ This shows that these catalysts are promising for esterification reactions since linker nitrogen sites allow variable Brønsted acidity while MOF confinement stops acid leaching. As with the preceding examples, esterification of isooctyl alcohol with acetic acid is a model reaction; its relevance here lies in demonstrating that linker-based Brønsted acidity can be tuned without framework degradation.

**Fig. 9:**
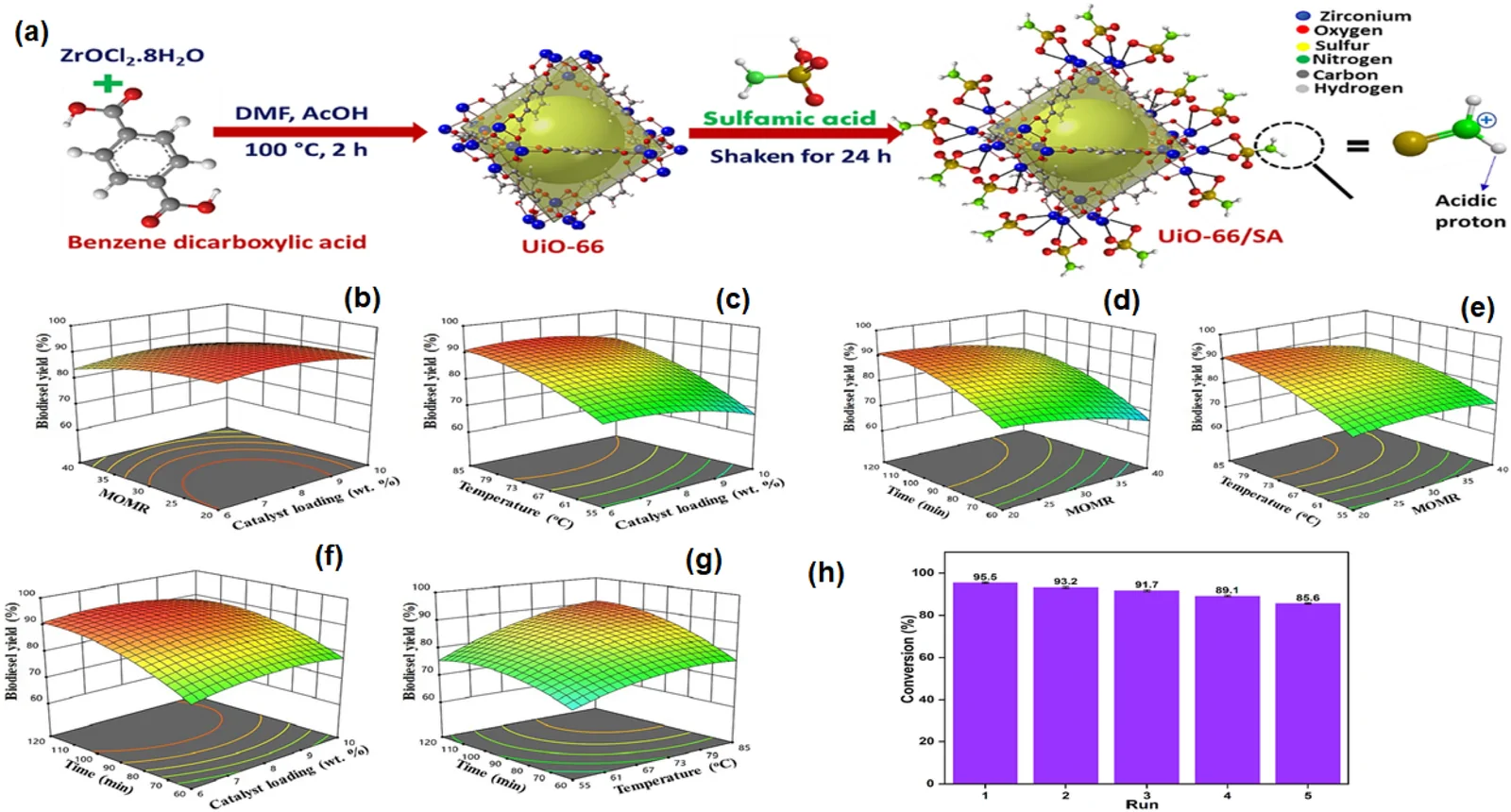
(a) Schematic illustration of UiO-66/SA synthesis *via* one-pot method followed by sulfamic acid functionalization. (b–g) 3D response surface plots showing the effect of (b) methanol-to-oleic-acid molar ratio (MOMR) *vs.* catalyst loading, (c) temperature *vs.* catalyst loading, (d) time *vs.* MOMR, (e) temperature *vs.* MOMR, (f) time *vs.* catalyst loading, and (g) time *vs.* temperature on biodiesel yield. (h) Reusability study showing oleic acid conversion over 5 cycles. Reaction conditions: optimum MOMR 21.8 : 1, catalyst loading 7.6 wt%, *T* = 85 °C, *t* = 1.8 h.^74^ This figure has been adapted/reproduced from ref. 74 with permission from Elsevier, copyright 2024.

By treating UiO-66 with sulfamic acid to provide Brønsted acidic –SO_3_H groups for esterification of oleic acid with methanol, linker functionalisation was successfully accomplished. The catalyst was made *via* one-pot synthesis of UiO-66 and post-synthetic modification with sulfamic acid to improve acidic site density, as shown in Fig. 9a. Fig. 9a–g display the synthesis method and RSM-CCD optimization plots, while Fig. 9h shows catalyst stability across five cycles. The 3D surface plots in Fig. 9b–g illustrate the ideal parameters of methanol to oleic acid molar ratio 21.8 : 1, catalyst loading 7.6 weight percent, temperature 85 °C, and time 1.8 hours that were obtained using response surface methods based on central composite design. With an acidity of 1.4 mmol g^−1^, the sulfamic acid-modified UiO-66 demonstrated remarkable catalytic activity under these circumstances, yielding 94.4 ± 0.3% of biodiesel and converting 95.5 ± 0.3% of oleic acid. As shown in Fig. 9h, the catalyst was recycled five times with oleic acid conversion falling from 95.5 ± 0.3% to 85.6 ± 0.2%, a decline the authors attribute to slight leaching of active sites during washing and reactivation, evidenced by reduced sulfur and nitrogen content in the recovered catalyst (CHNS) and by weakened XRD and FT-IR signals. A hot-filtration test nevertheless confirmed that the catalysis is genuinely heterogeneous: conversion stalled at 85.1% on removal of the catalyst and reached only 85.4% after a further 60 min. Linker functionalization is a successful tactic for improving esterification performance, as demonstrated by RSM-CCD analysis, which showed that reaction temperature and time were the most sensitive factors influencing biodiesel output. The esterification proceeded with an activation energy of 41.6 kJ mol^−1^ and was confirmed thermodynamically to be endothermic and non-spontaneous.^74^

**Fig. 10:**
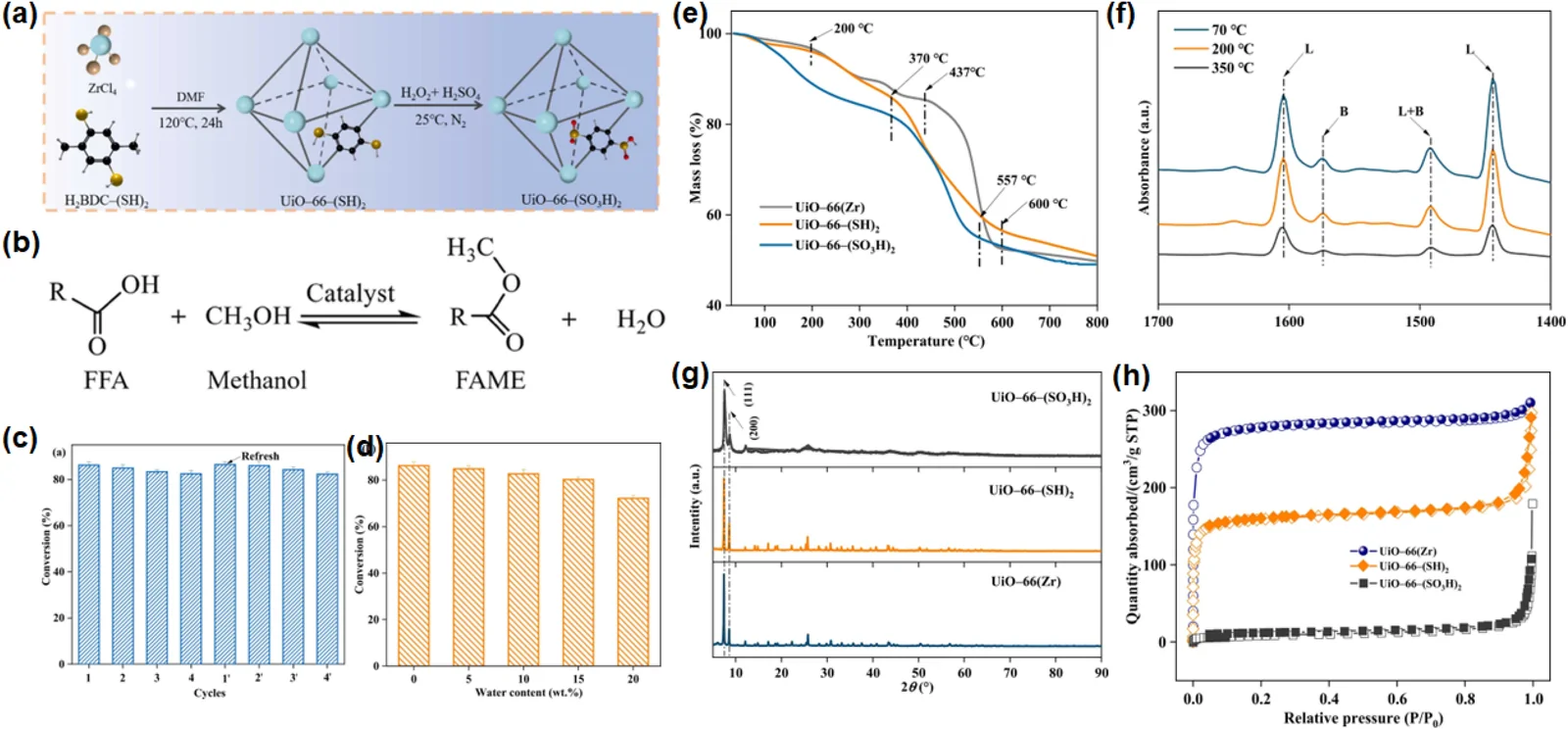
(a) Synthesis route of UiO-66-(SO_3_H)_2_*via* linker modification with 2,5-dimercaptoterephthalic acid and H_2_O_2_ oxidation. (b) Esterification reaction of free fatty acids with methanol. (c) Catalyst reusability over four cycles, followed by regeneration and four further cycles. (d) Effect of water content on conversion. (e) TGA curves, (f) Py-IR spectra showing Lewis L and Brønsted B acid sites, (g) XRD patterns, and (h) N_2_ adsorption–desorption isotherms of UiO-66, UiO-66-(SH)_2_, and UiO-66-(SO_3_H)_2_.^75^. This figure has been adapted/reproduced from ref. 75 with permission from Elsevier, copyright 2024.

Li *et al.*^75^ functionalized UiO-66 using 2,5-dimercaptoterephthalic acid as the linker, followed by *in situ* oxidation of the –SH groups with H_2_O_2_ to generate –SO_3_H groups, affording UiO-66-(SO_3_H)_2_ (Fig. 10a); the resulting material was applied to the esterification of free fatty acids with methanol to produce FAME (Fig. 10b). This post-synthetic oxidation raised the Brønsted acidity from 0.02 mmol g^−1^ in UiO-66-(SH)_2_ to 2.28 mmol g^−1^ in UiO-66-(SO_3_H)_2_. Thermogravimetric analysis (Fig. 10e) showed a modest reduction in thermal stability relative to pristine UiO-66(Zr), yet the functionalized framework remained stable well beyond the temperatures required for biodiesel synthesis. PXRD (Fig. 10g) retained the characteristic (111) and (200) reflections at 7.34° and 8.48°, confirming that the UiO-66 topology survived linker modification, while the reduced peak intensity indicates a higher defect density and hence a greater number of accessible active sites. Pyridine-IR spectra (Fig. 10f) confirmed the coexistence of Lewis acid sites at the coordinatively unsaturated Zr nodes and Brønsted acid sites at the –SO_3_H groups. N_2_ sorption (Fig. 10h) revealed that the bulky –SH and –SO_3_H substituents reduce both BET surface area and pore volume, but the type IV isotherms with H3 hysteresis confirm the development of mesopores exceeding 10 nm; this added mesoporosity improves mass transfer of bulky triglyceride molecules and thereby accelerates esterification kinetics. Of greatest practical significance, UiO-66-(SO_3_H)_2_ gave a maximum oleic acid conversion of 86.5% (10 wt% catalyst, 15 : 1 methanol : oleic acid, 90 °C, 4 h) and lost only 3.5 percentage points over four consecutive cycles, with activity fully restored by washing in cyclohexane and ethanol followed by re-acidification in dilute sulfuric acid (Fig. 10c). A hot-filtration test confirmed genuine heterogeneity, the filtrate giving only 3.0% conversion while the recovered solid gave 86.0%. Water resistance is the more consequential result: conversion was 84.9% at 5 wt% and 82.7% at 10 wt% added water, falling by 6.6 percentage points only at 20 wt%, where partial hydrolysis of the –SO_3_H groups sets in against a 76% collapse reported for sulfuric acid under the same water loading (Fig. 10c) and tolerated water contents up to 15 wt% with only modest loss of conversion (Fig. 10d). Taken together, these results show that linker functionalization, whether by grafting, impregnation, or loading of acidic species provides a direct route to tuning the acid site density of Zr-MOFs for improved biodiesel catalysis.

### 2.5 Composite formations

The stability, acidity, and recoverability of Zr-MOFs for biodiesel synthesis are improved by composite creation with metal oxides, heteropolyacids (HPAs), and magnetic nanoparticles.

#### 2.5.1 Metal oxide composites for waste oil

Coupling Zr-MOFs to metal oxides introduces additional active sites while preserving the porosity of the framework. Maleki *et al.* prepared a UiO-66-NH_2_/ZnO/TiO_2_ nano-composite by a sol–gel route followed by *in situ* growth of the MOF, and applied it to the transesterification of dairy waste scum oil (DWSO), an abundant and inedible waste stream from the dairy industry.^76^ Since the acid value of the DWSO was low—the authors report it as below 1.24%, determined by acid–base titration—no pre-esterification step was required, and the feedstock could be transesterified directly. CO_2_-TPD confirmed that incorporation of ZnO and TiO_2_ progressively strengthened the basicity of the framework, the total density of basic sites rising from 0.743 mmol g^−1^ in pristine UiO-66-NH_2_ to 0.908 mmol g^−1^ in UiO-66-NH_2_/TiO_2_ and 1.214 mmol g^−1^ in the ternary composite. This gain in basicity comes at the expense of surface area, which fell from 971 to 568 m^2^ g^−1^ as the oxides occupied the pores, although the mean pore diameter increased from 2.43 to 3.52 nm, preserving the mesoporosity required for bulky triglyceride diffusion. Under ultrasonic irradiation and conditions optimized by response surface methodology based on a central composite design (RSM-CCD) optimized conditions—a methanol-to-DWSO molar ratio of 9.8 : 1, 61 °C, 2 wt% catalyst, and 30 min of sonication—the ternary composite achieved 98.7% conversion to methyl esters, exceeding both UiO-66-NH_2_/TiO_2_ (96.8%) and pristine UiO-66-NH_2_ (96.1%) and confirming the synergistic contribution of the two oxides. Kinetic analysis gave an activation energy of 48.7 kJ mol^−1^, and the thermodynamic parameters (Δ*H* = +46.0 kJ mol^−1^; Δ*G* = +88.9 kJ mol^−1^ at 338 K) indicate an endothermic, non-spontaneous process. The catalyst retained 90.1% conversion after seven consecutive cycles, and blending the resulting biodiesel into diesel improved compression–ignition engine power while lowering CO and unburned hydrocarbon emissions, demonstrating the practical viability of metal oxide-MOF composites for valorizing waste streams.

#### 2.5.2 Heteropolyacid (HPA) composites for strong acidity

Heteropolyacids such as H_3_PW_12_O_40_ possess Brønsted acidity exceeding that of sulfuric acid, but their low surface area and solubility in polar media cause leaching and effectively homogeneous behaviour. Both problems can be mitigated by partial substitution of the acidic protons with large monovalent cations and by encapsulation within a porous host. Zhang *et al.* combined the two strategies, first preparing the ammonium- and silver-co-doped phosphotungstate Ag_1_(NH_4_)_2_PW_12_O_40_ (ref. 77) and then encapsulating it within UiO-66 by a one-pot solvothermal route to give Ag_1_(NH_4_)_2_PW_12_O_40_/UiO-66.^78^ FT-IR and PXRD confirmed that both the Keggin structure and the UiO-66 topology were retained, while the absence of discrete heteropolyacid reflections in the composite indicated uniform dispersion of the salt within the framework cavities. N_2_ sorption showed that the isotherm character of UiO-66 was preserved, although the BET surface area decreased from 667 to 555 m^2^ g^−1^ and the pore volume from 0.43 to 0.34 cm^3^ g^−1^, consistent with partial occupation of the pores by the guest. NH_3_-TPD was reported to show a large population of moderately strong sites desorbing between 150 and 300 °C alongside weak sites below 150 °C; the quoted densities (11.7 and 1.5 mmol g^−1^) are high relative to other Zr-MOF acid catalysts surveyed here and are reproduced as published. In the esterification of lauric acid with methanol, the composite achieved 75.6% conversion under optimized conditions (10 wt% catalyst, lauric acid : methanol 1 : 15, 150 °C, 3 h), following pseudo-first-order kinetics with an activation energy of 35.2 kJ mol^−1^. The value of encapsulation emerges from comparison with the unsupported salt, though the two studies are not directly comparable. Mesoporous Ag_1_(NH_4_)_2_PW_12_O_40_ alone converts oleic acid to 96.1% at 70 °C in 4 h at a 1 : 10 acid-to-methanol ratio with 250 mg catalyst,^77^ whereas the composite requires 150 °C, a 1 : 15 ratio and 3 h to reach 75.6% on lauric acid. Encapsulation therefore does not enhance intrinsic activity; the guest occupies part of the pore network, the BET surface area falling from 667 to 555 m^2^ g^−1^ and the pore volume from 0.43 to 0.34 cm^3^ g^−1^ on incorporation, and the accessible proton density falls accordingly. What is gained is operational stability. The free salt retains only 56.0% conversion after four cycles, whereas the composite holds 70.6–75.6% across the same number, the decline to 54.6% appearing only at the sixth cycle and attributable to both gradual loss of active species and accumulation of surface-bound lauric acid and methyl laurate that methanol washing does not remove. Confinement within the framework thus retards, without eliminating, deactivation of the Keggin phase. The catalysis is bifunctional in origin: Brønsted protons remain on the partially substituted Keggin anion, supplying the strong acidity that drives esterification, while the Lewis acidic Zr^4+^ centres of the UiO-66 node polarise the carboxyl group of the fatty acid. The NH_3_-TPD profile is consistent with this, showing weak sites below 150 °C (1.5 mmol g^−1^) alongside a much larger population of moderately strong sites between 150 and 300 °C (11.7 mmol g^−1^). Two questions remain open in this system, however: no catalytic data are reported for UiO-66 in the absence of the salt, so the framework's own contribution to activity cannot be separated from that of the guest, and the composite was prepared at a single salt loading, leaving the trade-off between Brønsted acid density and pore blockage uncharacterised.^78^

A complementary architecture embeds the heteropolyacid within a secondary MOF before incorporation into the Zr framework. Zhang *et al.* immobilized silicotungstic acid (HSiW) on a Ce-based MOF (Ce-BDC) and embedded the resulting HSiW@Ce-BDC within UiO-66(Zr), affording Ce-BDC@HSiW@UiO-66.^79^ NH_3_-TPD confirmed a higher acid strength than HSiW@Ce-BDC alone, arising from the additional Lewis acidity of the surface Zr^4+^ centers. Under mild conditions (0.2 g catalyst, oleic acid : methanol 1 : 30, 130 °C, 4 h), the hybrid achieved 81.5% oleic acid conversion, compared with only 21.9% for HSiW@Ce-BDC in the absence of the Zr framework. The difference persists on reuse: HSiW@Ce-BDC remains near 20% conversion by its third cycle, whereas the composite loses only 4.6% of its initial conversion over six, and a hot-filtration test in which the catalyst was removed after 2 h produced no further conversion, confirming that the catalysis is genuinely heterogeneous. Two effects account for the improvement. The first is textural. Incorporation of the guest converts the type I isotherm of microporous UiO-66 into a type IV isotherm with a hysteresis loop, and although the BET surface area falls from 667 to 251 m^2^ g^−1^ and the pore volume from 0.43 to 0.22 cm^3^ g^−1^, the mean pore diameter rises from 2.6 to 3.4 nm with a distinct population near 3.9 nm. That conversion improves while surface area falls by more than half indicates that total area is not the limiting variable here, though the comparison is between two different materials whose acid-site inventories also differ, so pore accessibility cannot be isolated as the sole cause. The second is the acid-site inventory. NH_3_-TPD of HSiW@Ce-BDC shows desorption at 113 and 570 °C, the latter reflecting the strong Brønsted acidity of the silicotungstate; in the composite this becomes a sharper feature at 579 °C with a shoulder at 545 °C, the shoulder being attributed to Lewis acidic Zr^4+^ centers introduced with the framework. Brønsted activation of methanol and Lewis polarization of the fatty acid carboxyl therefore occurs at neighboring sites. The substrate scope is less easily rationalized. Under identical conditions the catalyst converts lauric acid to 56.8%, myristic acid to 79.8%, palmitic acid to 96.3%, stearic acid to 77.3% and oleic acid to 81.5%, a non-monotonic dependence on chain length that a simple diffusional argument does not account for, since the shortest substrate performs worst. The original authors offer no mechanistic explanation, and none is available from the data reported; the series is noted here because it illustrates how rarely substrate-scope results in this literature are interpreted rather than tabulated. Two limitations should be noted. No catalytic data are reported for UiO-66 or Ce-BDC alone, so the framework's individual contribution cannot be isolated, and the composite was prepared at a single HSiW loading, leaving the balance between Brønsted acid density and pore blockage uncharacterized. A leaching test confirmed the heterogeneous nature of the catalysis, and the composite was reused six times with a decline in conversion of only 4.6%.^79^

**Fig. 11:**
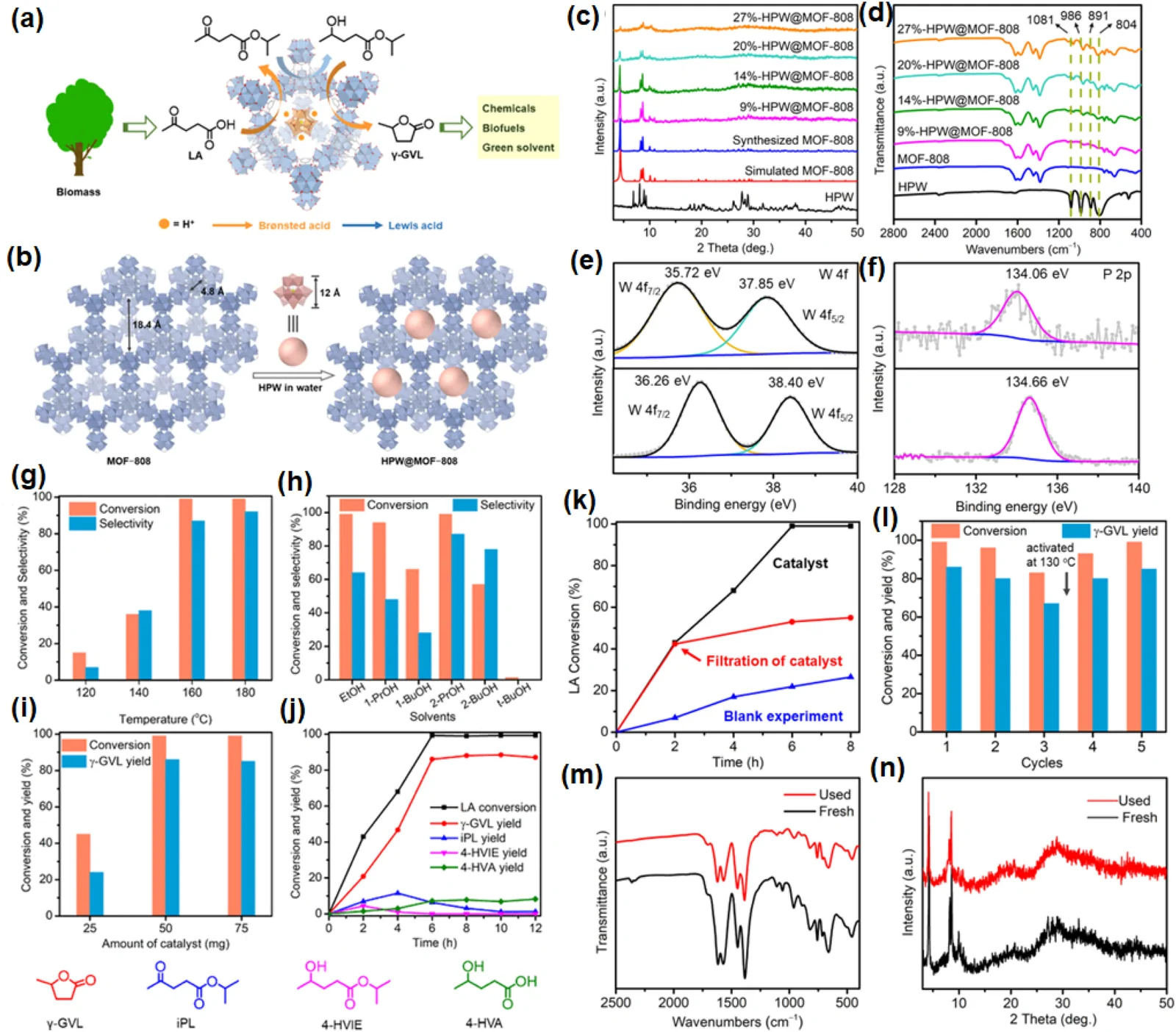
(a) Schematic of biomass-derived levulinic acid conversion to γ-GVL *via* esterification–lactonization over HPW@MOF-808, showing Brønsted H^+^ and Lewis Zr^4+^ sites. (b) Structure of MOF-808 with 18.4 Å and 4.8 Å cavities for HPW encapsulation. (c) PXRD patterns, (d) FTIR spectra, (e,f) XPS spectra of W 4f and P 2p. (g–j) Catalytic performance: effect of temperature, solvent, catalyst amount, and time course. (k) Hot filtration test. (l) Reusability over 5 cycles. (m, and n) FTIR and PXRD of fresh *vs.* used catalyst.^80^ This figure has been adapted/reproduced from ref. 80 with permission from the American Chemical Society, copyright 2021.

MOF-808 possesses 6-connected Zr_6_ clusters, in contrast to the 12-connected clusters of UiO-66 and therefore offers a higher density of coordinatively unsaturated Zr^4+^ sites together with substantially larger pores. It nevertheless lacks sufficient Brønsted acidity to drive esterification–lactonization cascades. This limitation is overcome by pairing the Lewis acidic Zr^4+^ nodes with Brønsted acidic HPW, achieved by impregnating H_3_PW_12_O_40_ into MOF-808.^80^ Impregnation was used to create HPW@MOF-808 (Fig. 11a and b). The question that matters here is whether the Keggin anion sits inside the framework cavities or merely on the external surface, since only confinement prevents leaching. Three observations point to confinement. The MOF-808 reflections weaken progressively with loading rather than being joined by separate HPW reflections (Fig. 11c), indicating dispersion rather than a second phase; the W=O and W–O–W bands persist in the composite (Fig. 11d), showing the Keggin anion survives impregnation intact; and the 18.4 Å cavity of MOF-808 (Fig. 11b) is large enough to accommodate a Keggin cluster of about 1 nm. Retention of both W^6+^ and Zr^4+^ (Fig. 11e and f) confirms neither component is reduced during synthesis, so Brønsted and Lewis sites remain simultaneously available. Confinement is proved functionally, however, by the hot-filtration test and the absence of HPW leaching over five cycles (Fig. 11k and l). 14%-HPW@MOF-808 produced an 86% GVL yield when levulinic acid LA was converted to γ-valerolactone GVL in isopropanol (Fig. 11g–j). Both the esterification and lactonization processes were catalyzed by the combination of Lewis Zr^4+^ si tes of MOF-808 and Brønsted –OH of HPW. After five cycles with no HPW leaching, post-catalysis PXRD/FTIR in Fig. 11k–n verified structural integrity, and SEM-EDS mapping revealed a uniform polyoxometalate (POM) distribution. For bulky LA in mild conditions, MOF-808's wide surface area and open voids allowed for quick mass transfer. Levulinic acid is a platform molecule of independent interest, its esters and γ-valerolactone having been developed as fuel additives and blending components.^71,81–83^ Although the levulinic acid-to-GVL cascade is not itself a biodiesel transformation, it probes the same Brønsted–Lewis acid pairing that governs esterification of free fatty acids, and the HPW@MOF-808 design principal transfers directly to triglyceride and FFA conversion.

Keggin polyoxometalates (POMs) are encapsulated in Zr-MOFs to maintain robust acidity while preventing leaching. NiHSiW/UiO-66 was created by encasing Ni ions of silicotungstic acid H_4_SiW in UiO-66 using one-pot hydrothermal synthesis. Over the course of eight consecutive trials, the composite produced 86.7% biodiesel conversion for oleic acid esterification. PXRD verified that the Keggin structure remained intact upon reuse. The high activity arises from the retained porosity of the framework, the dispersion of NiHSiW nanoparticles, and the synergistic action of NiHSiW acid sites with the UiO-66 matrix. With *E*_a_ = 69.2 kJ mol^−1^, kinetics were pseudo-first-order.^84^

Similarly, HPW@UiO-67 for *n*-butyl acetate production was created by hydrothermally confining phosphotungstic acid in size-matched UiO-67 cages. The ∼1.8 nm octahedral cage of UiO-67 accommodates the ∼1.2 nm Keggin polyanion, so encapsulation proceeds without disrupting the framework; PXRD showed no free HPW reflections, and N_2_ sorption confirmed the guest occupies the micropores, the BET area falling from 2259 to 1023 m^2^ g^−1^ and the micropore volume from 0.79 to 0.34 cm^3^ g^−1^ while the pore distribution remained at 1.0–2.0 nm. Total acidity rose from 0.362 to 0.504 mmol g^−1^. The composite gave 72.3% acetic acid conversion in 6 h at 120 °C, following second-order kinetics with *E*_a_ = 53.1 kJ mol^−1^, and retained 63.1% conversion after four cycles. Matching the cavity dimension to the heteropolyanion is what confers this stability.^85^

#### 2.5.3 Metal nanoparticle composites

**Fig. 12:**
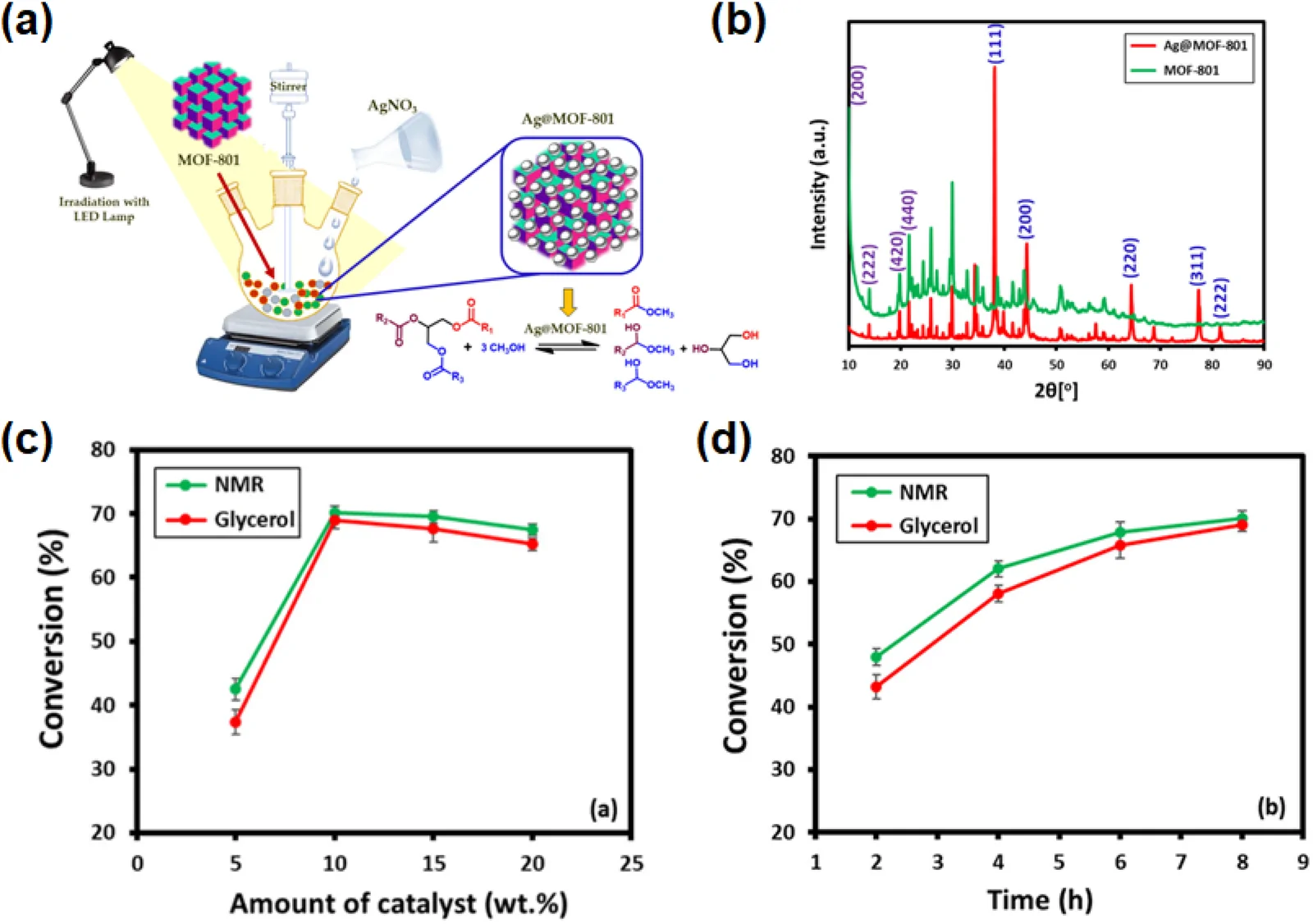
(a) Visible-light photoreduction synthesis of Ag@MOF-801 and transesterification of triglycerides to FAME + glycerol. (b) XRD patterns of MOF-801 and Ag@MOF-801 showing Ag (111), (200), (220), (311) peaks. (c) Effect of catalyst loading and (d) reaction time on conversion measured by ^1^H NMR and glycerol yield. Optimal: 10 wt% catalyst, 8 h.^86^ This figure has been reproduced from ref. 86 under a Creative Commons CC-BY license, copyright 2022 the authors, published by MDPI.

Zr^4+^ nodes of MOF-801 were used to decrease and deposit Ag NPs on the surface of Ag@MOF-801 using visible light photoreduction (Fig. 12a). XRD shows the (111), (200), (220) and (311) reflections of metallic silver on an otherwise unchanged MOF-801 pattern (Fig. 12b), establishing that photoreduction produces discrete Ag^0^ nanoparticles rather than exchange of Ag^+^ into the framework. This matters mechanistically: the modest gain over pristine MOF-801 (69% *versus* 60%) therefore comes from the plasmonic Ag^0^ particles and the increased surface area, not from any change at the Zr node. That conversion rises to 73.1% only when HCl is added confirms Brønsted acidity remains the limiting factor. Glycerol production was confirmed by NMR in Fig. 12c and d, and the ideal conditions were 10% catalyst and 8 hours.^86^

#### 2.5.4 Magnetic composites for catalyst recovery

**Fig. 13:**
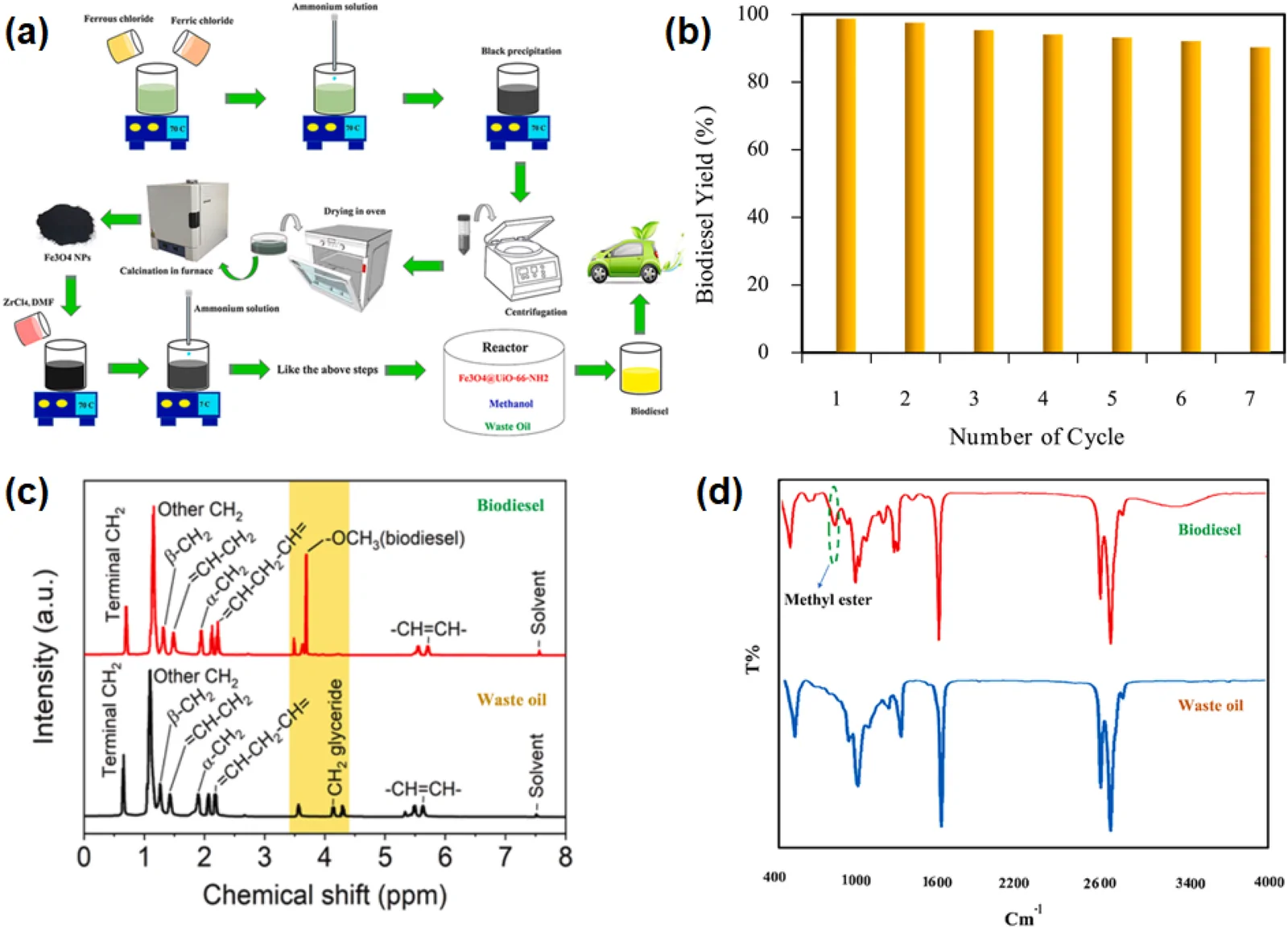
(a) Schematic illustration of Fe_3_O_4_@UiO-66-NH_2_ synthesis *via* co-precipitation of Fe_3_O_4_ followed by *in situ* growth of UiO-66-NH_2_. (b) Reusability study showing biodiesel yield over 7 cycles using restaurant waste oil. (c) ^1^H NMR spectra and (d) FTIR spectra of waste oil and biodiesel showing disappearance of glyceride peaks and appearance of methyl ester peaks at 3.7 ppm and 1739 cm^−1^. Reaction conditions: microwave irradiation, 8.8 min, 98.2% purity.^87^ This figure has been adapted/reproduced from ref. 87 with permission from Elsevier, copyright 2024.

Fe_3_O_4_@UiO-66-NH_2_ was prepared by Sun *et al.* through co-precipitation of Fe_3_O_4_ nanoparticles followed by *in situ* growth of UiO-66-NH_2_ on the magnetic core, as illustrated schematically in Fig. 13a.^87^ Ferrous and ferric chloride were co-precipitated with ammonia to form Fe_3_O_4_, which was then combined with ZrCl_4_ and 2-aminoterephthalic acid in DMF so that the MOF shell nucleated directly on the oxide surface. This architecture couples the Lewis acidic Zr^4+^ nodes and Brønsted basic –NH_2_ groups of UiO-66-NH_2_ to a magnetically responsive core, allowing the catalyst to be recovered from the reaction mixture with an external magnet rather than by filtration or centrifugation. Successful transesterification of restaurant waste oil was confirmed spectroscopically. The ^1^H NMR spectra in Fig. 13c show the appearance of the characteristic –OCH_3_ singlet of the methyl ester at 3.7 ppm in the product, accompanied by the disappearance of the glyceride –CH_2_ resonances present in the parent waste oil, indicating essentially complete conversion of triglycerides to FAME. The FTIR spectra in Fig. 13d corroborate this, with a new C=O ester stretch emerging at 1739 cm^−1^ and a marked attenuation of the broad –OH band between 3100 and 3600 cm^−1^, consistent with consumption of free fatty acids and glycerol-bound hydroxyls.

Under microwave irradiation and RSM-CCD optimized conditions, the nanocomposite delivered 98.2% biodiesel purity in only 8.8 min, a reaction time far shorter than that typical of conventional heating, reflecting the efficient dielectric heating of the magnetite core. The contrast with conventionally heated systems is instructive. Thermally driven esterification and transesterification over the Zr-MOF catalysts surveyed here require hours rather than minutes: sulfamic-acid-modified UiO-66 needs 1.8 h at 85 °C,^74^ the heteropolyacid composites 3 h at 150 °C^78^ and 4 h at 130 °C,^79^ and the lipase-functionalized Zr-MOF/PVP membrane 12 h.^88^ Microwave irradiation reduces the same transformation to under 9 min. The reason lies in how the energy is delivered. Conventional heating must transfer energy through the vessel wall and then through a poorly conducting, largely non-polar oil phase, whereas microwave energy is absorbed directly by polar methanol and, in this system, by the magnetite core, which couples strongly to the field and generates heat at the catalyst surface where the reaction occurs. The rate-limiting resistance in triglyceride conversion is thus relieved at its origin rather than compensated for by prolonged heating. Ultrasound achieves a comparable acceleration by a different mechanism: the UiO-66-NH_2_/ZnO/TiO_2_ composite reaches 98.7% conversion in 30 min at 61 °C,^76^ cavitation generating the interfacial area needed to bring the immiscible oil and methanol phases into contact. Both forms of process intensification therefore act on mass transfer rather than on the intrinsic acidity of the catalyst, which is why they combine so effectively with magnetic and mesoporous framework designs the modification supplies accessible active sites, and the energy input ensures the substrate reaches them. The reusability data in Fig. 13b show that the catalyst retained biodiesel yield above 90% across seven consecutive cycles, with only a gradual decline attributable to minor mass loss during magnetic separation rather than to framework degradation. Kinetic analysis indicated an endothermic reaction with a temperature-dependent rate constant, and engine tests on diesel–biodiesel blends produced with Fe_3_O_4_@UiO-66-NH_2_ showed improved compression–ignition performance and reduced emissions. Taken together, these results demonstrate that magnetic composite formation addresses the two practical bottlenecks of powdered Zr-MOF catalysts—separation difficulty and mechanical loss on recovery—without sacrificing the acid–base bifunctionality that drives the transesterification itself.

### 2.6 Enzyme immobilization

Lipase-catalysed transesterification offers high selectivity under mild conditions, but the operating window of the free enzyme is narrow and understanding why explains what immobilisation on a Zr-MOF is for. Lipases operate optimally between about 30 and 50 °C, and although some tolerate excursions towards 70 °C, prolonged exposure above this range unfolds the tertiary structure and destroys the active site, so the reaction cannot simply be accelerated by heating as it can over a solid acid.

The chemical environment of biodiesel synthesis is more damaging still, and three distinct mechanisms operate. The first is alcohol toxicity. Short-chain alcohols, methanol especially, are sufficiently polar to strip the layer of hydration water that maintains the folded conformation of the enzyme, and the resulting structural collapse is irreversible.^89^ The second is a consequence of phase behaviour rather than chemistry: methanol is only partially miscible with triglyceride oils, so above its solubility limit it separates as droplets that adhere to the enzyme particles and expose them to a locally undiluted alcohol phase, causing rapid and permanent loss of activity.^89,90^ Stepwise methanol addition, or the use of ethanol or an acyl acceptor such as methyl acetate, is the usual mitigation.^90^ The third arises from the product side. Glycerol is hydrophilic and viscous and being poorly soluble in the oil phase it accumulates as a film on the support surface, physically obstructing access of the triglyceride substrate to the active site.^91^ Because the effect is a transport barrier rather than a chemical one, activity can often be partially restored by washing the recovered biocatalyst.

**Fig. 14:**
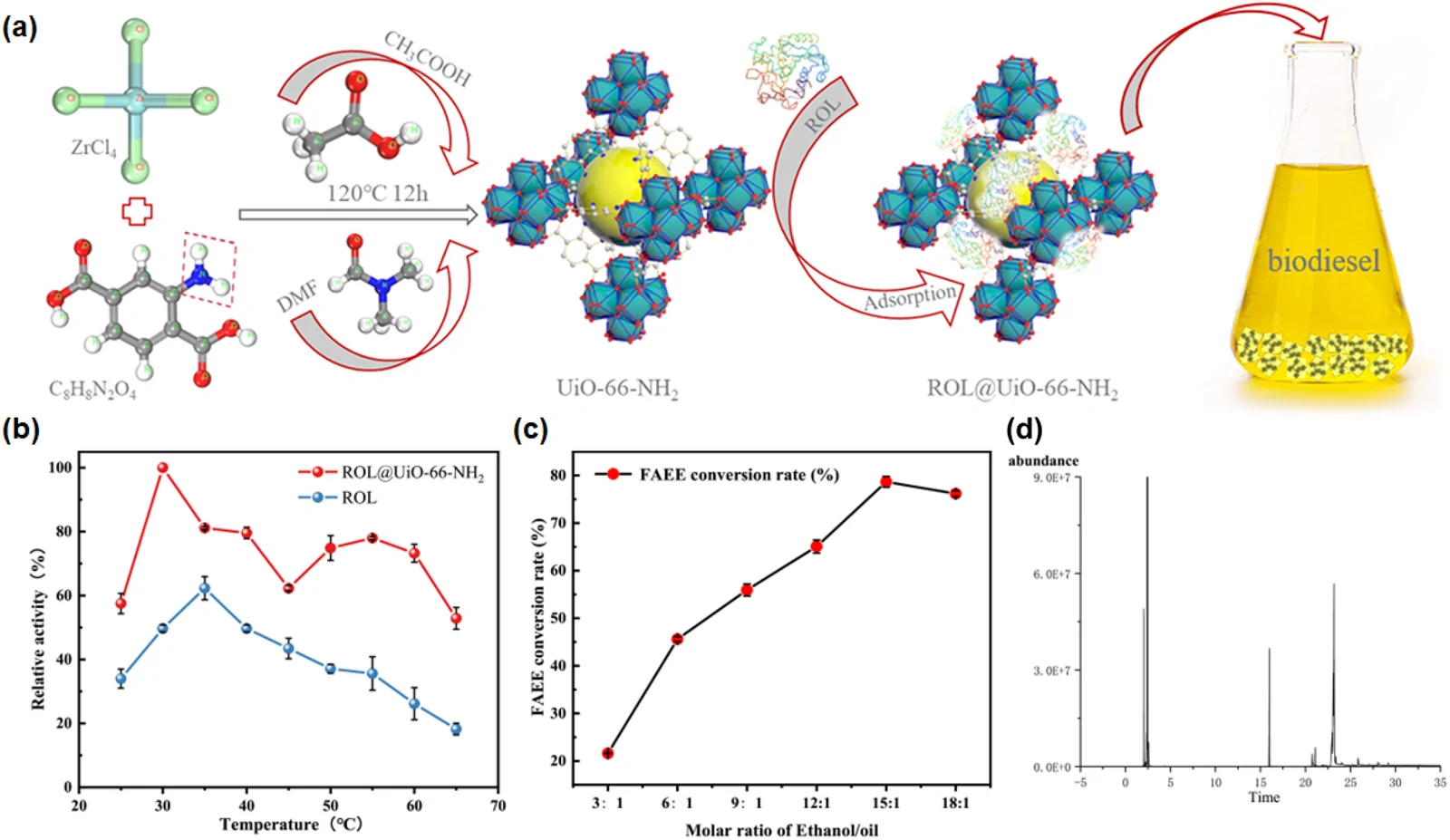
(a) Synthesis of UiO-66-NH_2_ from ZrCl_4_ and 2-aminoterephthalic acid with acetic acid modulator, followed by physical adsorption of Rhizopus oryzae lipase ROL to form ROL@UiO-66-NH_2_ for biodiesel production. (b) Temperature effect on relative activity of free ROL *vs.* immobilized ROL@UiO-66-NH_2_. (c) Effect of ethanol/oil molar ratio on FAEE conversion rate. (d) GC-MS chromatogram of FAEE products from waste oil.^92^ This figure has been adapted/reproduced from ref. 92 with permission from Elsevier, copyright 2024.

Immobilization on a Zr-MOF addresses all three. The framework anchors the enzyme in a defined conformation so that partial dehydration by the alcohol no longer leads to collapse; its porosity and surface chemistry moderate the local alcohol concentration reaching the protein; and the rigid, recoverable support allows glycerol to be removed between cycles without loss of the biocatalyst. These are the mechanisms that account for the improvements in thermal and operational stability described below. The thermal stability and reusability of lipases to produce biodiesel are enhanced by enzyme immobilization on MOFs. As shown in Fig. 14a, *Rhizopus oryzae* lipase ROL was immobilized on UiO-66-NH_2_*via* interfacial adsorption. ROL was adsorbed through H-bonding with –NH_2_ groups after UiO-66-NH_2_ was created from ZrCl_4_ and 2-aminoterephthalic acid using acetic acid as a modulator at 120 °C for 12 hours. Immobilized ROL@UiO-66-NH_2_ maintained 73.2% relative activity at 60 °C, approximately 35% higher than free ROL, and remained stable up to 65 °C, according to thermal stability studies in Fig. 14b, demonstrating that the MOF framework maintains enzyme conformation. Fig. 14c illustrates how FAEE conversion for ethanolysis of waste oil grew with the ethanol/oil molar ratio, reaching 78.7% at 15 : 1, whereas RSM optimization produced 82.1% conversion. After five cycles, the catalyst's conversion rate remained at 71.9%. FAEE products with C_14_–C_24_ chains, primarily ethyl laurate, ethyl oleate, and ethyl linoleate, were confirmed by GC-MS in Fig. 14d. Immobilization comes at a textural cost: the BET surface area of UiO-66-NH_2_ falls from 739 to 522 m^2^ g^−1^ and the pore diameter from 5.3 to 3.4 nm as the protein occupies the pore network. The immobilized enzyme demonstrated superior heat resistance and pH tolerance compared with free ROL; micropore diffusion and methanol inhibition nevertheless remain obstacles to scale-up.^92^

Enzyme immobilization on Zr-MOFs addresses both the instability and the recovery of free lipases. Badoei-dalfard *et al.* developed a Zr-MOF/PVP electrospun nanofibrous membrane for the covalent immobilization of lipase MG10 from *Enterobacter cloacae*, applied to biodiesel production from *Ricinus communis* (castor) oil, a non-edible feedstock.^88^ Immobilization was optimized at 2.5% glutaraldehyde, 2 h crosslinking, and 6 h immobilization at pH 9.0, achieving an enzyme loading of up to ∼173 mg protein per gram of support. The immobilized biocatalyst substantially outperformed the free enzyme, reaching a biodiesel yield of ∼83% in 12 h, whereas the free lipase attained only 27% even after 18 h. Thermal stability improved markedly, by roughly 2.7-fold at 50 °C and 2.85-fold at 60 °C relative to the free enzyme. The immobilized lipase retained 92% of its initial activity after four reuse cycles and 66% after seven. The addition of divalent Cu^2+^ and Co^2+^ ions further enhanced activity, indicating a synergistic metal–MOF–enzyme interaction. Together these results show that the Zr-MOF/PVP nanofibrous support improves enzyme accessibility, thermal robustness, and operational stability, making it an effective platform for enzymatic biodiesel production.^88^

### 2.7 Cross-strategy comparison: which modification suits which feedstock

**Fig. 15:**
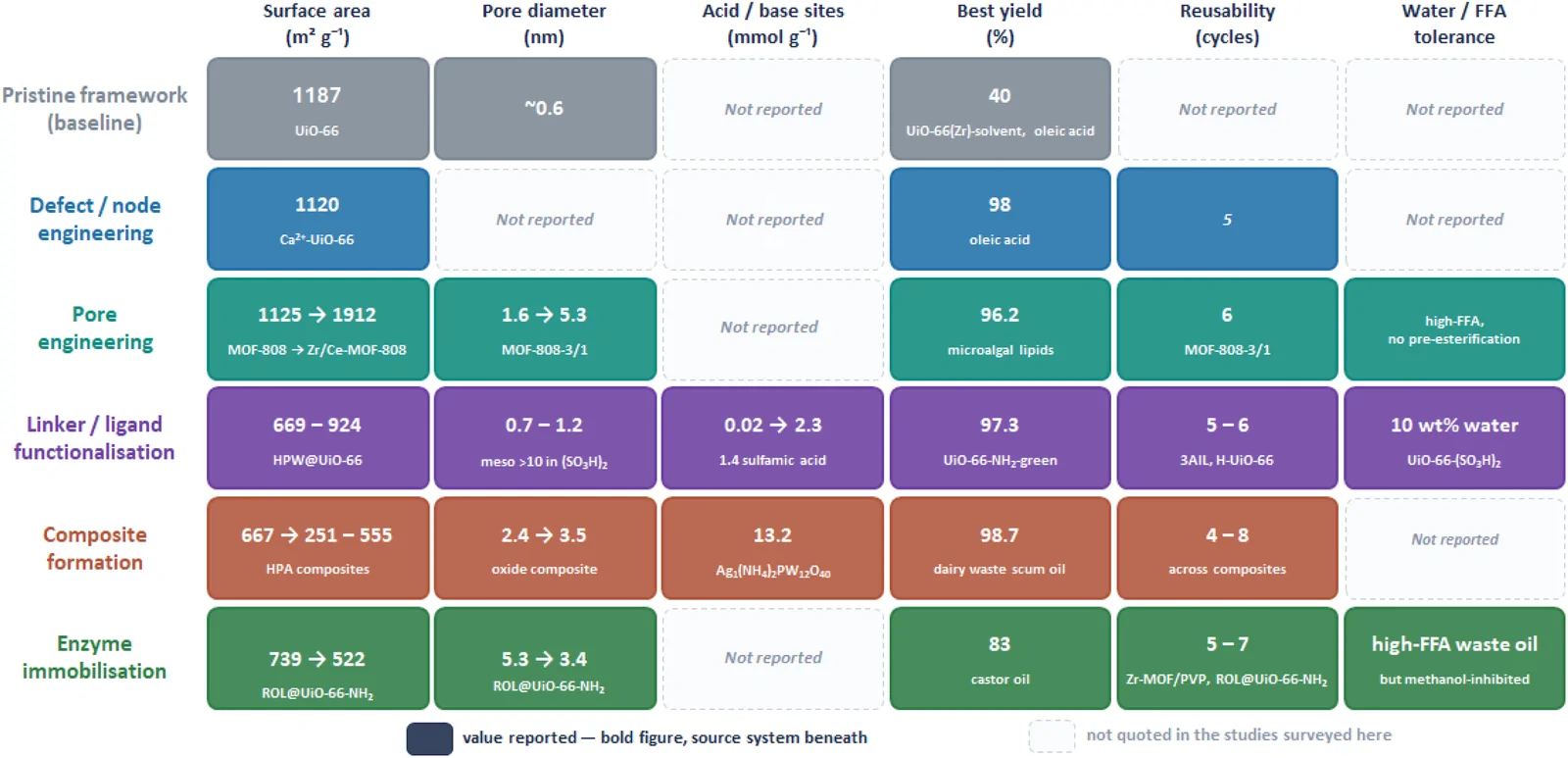
Comparison of the pristine framework and the five modification strategies across six catalyst descriptors. Each cell gives the value reported for the system named beneath it; dashed cells indicate parameters not quoted in those studies and should not be read as zero. The best-yield column is not a ranking – the entries correspond to different feedstocks, alcohols and temperatures. The one internally consistent comparison is the pristine baseline (40%) *versus* linker functionalization (97.3%), both measured for oleic acid esterification at 60 °C in the same study.^41^ Source citations appear in the corresponding sections.

Fig. 15 summarizes the five strategies against six descriptors and makes two features of the literature visible that are difficult to see study by study. The first is what is measured: of the thirty-six cells in the figure, eight are empty, and the pristine framework the baseline against which every modification is judged has been characterized for only three of the six descriptors. Reusability and water tolerance are reported far less often than surface area or yield, despite being the properties that determine whether a catalyst is usable at scale. The second is the direction of the trade-off. Pore engineering and bimetallic doping raise surface area (1125 to 1912 m^2^ g^−1^), whereas linker functionalization and enzyme immobilization lower it, the enzyme row falling from 739 to 522 m^2^ g^−1^ and the pore diameter from 5.3 to 3.4 nm as the protein occupies the pore network. Acid-site density moves in the opposite sense: sulfonation raises Brønsted acidity from 0.02 to 2.3 mmol g^−1^ while consuming pore volume. No single strategy improves both, and the choice between them is therefore governed by feedstock rather than by any general ranking of catalyst quality.

The five strategies surveyed above are not interchangeable, and the highest reported yield is a poor guide to which one to select. Before comparing them, it is worth stating plainly why a quantitative ranking is not available. Turnover frequency (TOF), the normalization that would be preferred, cannot be computed for this literature: it requires both the density of catalytically active sites and an initial rate measured under kinetic control, and most of the studies surveyed report neither. Where acid-site densities are given, they derive from NH_3_-TPD and count all sites titrated by ammonia rather than those participating in the catalytic cycle, so a TOF calculated from them would be a lower bound of unknown magnitude. Compounding this, TOF is itself used inconsistently in the catalysis literature, and a value derived from a single end-point yield rather than from kinetic measurement is an average rate over a changing composition rather than a property of the catalyst.^93^

Catalyst-normalized productivity, expressed here as grams of FAME per gram of catalyst per hour, is more tractable, since it requires only the yield, the catalyst loading and the reaction time, and it can be estimated for those studies reporting all three. Doing so is instructive. The ternary UiO-66-NH_2_/ZnO/TiO_2_ composite corresponds to roughly 98 g FAME per gram of catalyst per hour, sulfamic-acid-modified UiO-66 to about 7, Ag_1_(NH_4_)_2_PW_12_O_40_/UiO-66 to about 2.5, and Ag@MOF-801 to below 1. The reported yields of these four systems span 69–98%, a range that suggests broadly comparable performance; normalized for catalyst loading and time, they differ by two orders of magnitude, and the ordering is not the ordering of the yields. These figures are estimates and remain non-comparable in the strict sense, since the feedstocks, temperatures and alcohol ratios differ, and reaction time is an optimization endpoint rather than a kinetic measurement. They are given not as a ranking but as evidence that yield alone conveys almost no information about catalytic efficiency.

We therefore do not rank the strategies by performance. Meaningful benchmarking in catalysis requires standardized conditions and consistent reporting that this literature does not yet provide,^94^ and the appropriate response is to compare the strategies qualitatively, by the specific limitation each one relieves and the mechanistic basis on which it does so.

Defect and node engineering is the cheapest intervention and produces the largest relative gain, converting an essentially inactive defect-free UiO-66 into an active Lewis acid. However, it raises Lewis acidity without conferring Brønsted acidity, water tolerance, or resistance to free fatty acids. It is therefore best suited to refined, low-FFA oils, and reusability is seldom reported for defect-only catalysts.

Pore engineering addresses mass transfer rather than acidity. Enlarging apertures—as in the 6-connected MOF-808 framework—relieves diffusion limitations for bulky triglycerides and viscous feedstocks such as microalgal lipids but does not add active sites; MOF-808 in fact lacks the Brønsted acidity required for esterification cascades. Pore engineering is thus necessary but rarely sufficient and is most effective when combined with acid functionalisation.

Linker and ligand functionalisation deliver the highest acid-site density and is the only strategy for which tolerance to water and free fatty acids has been demonstrated directly; sulfonated UiO-66-(SO_3_H)_2_ loses only 3.5 percentage points of conversion over four cycles, recovers its initial activity after regeneration, and is essentially unaffected by water up to 10 wt%, degrading appreciably only at 20 wt% where the –SO_3_H groups begin to hydrolyse. This makes it the strategy of choice for high-FFA, wet waste feedstocks. Its cost is structural: bulky –SO_3_H or ionic–liquid moieties reduce BET surface area and pore volume, and unanchored acidic species are prone to leaching.

Composite formation contributes little to intrinsic activity but solves the two problems that most obstruct industrial deployment—catalyst recovery and Brønsted acid supply. Magnetic and metal-oxide composites achieve the highest yields reported on genuine waste feedstocks (98.2–98.7%) precisely because they combine strategies rather than relying on any single one, coupling acid–base bifunctionality to either magnetic separation or enhanced basicity.

Enzyme immobilisation operates under the mildest conditions and tolerates heterogeneous, low-grade feedstocks that deactivate thermal catalysts, but it consistently gives the lowest yields, the longest reaction times, and the poorest cycle stability, at the highest catalyst cost.

It is also worth situating Zr-MOFs against the solid acids they compete with. Sulfated zirconia is the closest comparator, since it exposes the same Zr^4+^ centres and reaches comparable conversions, but it suffers persistent leaching of sulfate under the polar conditions of esterification, which limits reuse. The advantage of the framework becomes clear when the two are compared directly: Li *et al.* impregnated UiO-66 with ammonium sulfate and found that anchoring the sulfate within the framework, rather than on bulk zirconia, gave 96.2% oleic acid conversion under mild conditions (8 wt% catalyst, methanol : oleic acid 8 : 1, 70 °C, 2 h) while markedly improving stability against active-site loss.^95^ Conventional solid bases such as CaO give high yields on refined oils but saponify with high-FFA feedstocks and leach calcium into the product; ion-exchange resins offer strong Brønsted acidity but are thermally limited and possess low surface area.^16,96^ Against these, the distinguishing feature of Zr-MOFs is not raw activity but the combination of Lewis acidity at the node with Brønsted acidity at terminal hydroxyls or grafted linkers within a single porous solid, allowing esterification and transesterification to proceed together and removing the separate acid pre-treatment step that low-grade feedstocks otherwise require.^14^

The central trade-off running through all these strategies is between acid-site density and pore accessibility. Grafting acidic groups increases the number of active sites but obstructs the channels through which triglycerides must reach them; enlarging pores improves access but lowers the volumetric density of active sites. The best-performing catalysts are those that decouple the two for example, by combining defect-induced hierarchical mesoporosity with post-synthetically grafted Brønsted acid sites, so that acidity is raised without paying the diffusional penalty. This design principle, rather than any single modification, is what distinguishes the most effective Zr-MOF biodiesel catalysts reported to date.

## 3 Future perspectives

Despite the progress reviewed above, several issues remain unresolved before Zr-MOF catalysts can be adopted industrially.

### 3.1 First, cost and scalability

Production costs have not been reported for any Zr-MOF biodiesel catalyst, and the few available MOF techno-economic analyses concern Al and Fe frameworks: MIL-160(Al) has been estimated at *ca.* 55 $ per kg at 100 t per year, falling to 29.5 $ per kg at 1 kt per year, and MIL-100(Fe) below 30 $ per kg when the synthetic route is chosen carefully. A published estimate for UiO-66-NH_2_ places it at 15.5 $ per kg at 2.5 kt per year, so Zr frameworks are not intrinsically more expensive at scale, though ZrCl_4_ and ZrOCl_2_·8H_2_O are appreciably more costly than the aluminium and iron salts on which those estimates rest,^97,98^ and since UiO-type syntheses are conventionally run in DMF rather than water. Against that baseline the five strategies separate clearly by cost driver rather than by absolute price. Defect engineering and linker functionalization are the least expensive, adding either nothing to the synthesis or a single substituted linker, and both have been demonstrated under solvent-free mechanochemical conditions that avoid DMF entirely. Linker cost can also be attacked from the feedstock side: terephthalate recovered from waste poly(ethylene terephthalate) has been used to build MIL-101(Cr) biodiesel catalysts at a fraction of the reagent cost,^24^ and since UiO-66 is built from the same BDC linker, the route transfers directly to Zr frameworks. Waste-derived and solvent-reduced routes to MIL-101(Cr) or MOFs have been developed on the same principle, indicating that precursor cost is a solvable problem rather than an intrinsic barrier.^99–103^ Pore engineering adds a modulator and, in the bimetallic case, a cerium salt. Composite formation spans a wide range: magnetite supports are inexpensive, whereas heteropolyacid and silver-containing composites carry tungsten and precious-metal costs that would dominate the catalyst price. Enzyme immobilization is the most expensive by a considerable margin, since commercial lipase costs exceed the cost of the support itself, and it is also the least compatible with scale-up given its methanol sensitivity and lower reusability. On present evidence, solvent-free linker functionalization is the only route that combines low precursor cost, green synthesis and high yield on genuine lipid feedstocks, and it is where scale-up effort is best directed.

### 3.2 Second, long-term stability under industrial conditions requires far more testing

Although many catalysts show good reusability over five to seven cycles, Zr leaching and ligand degradation in excess methanol and water could undermine economic viability over the far longer runs an industrial process demands. Strengthening the Zr–O bond through hydrophobic modification and controlled defect chemistry is essential, and stability should be assessed by post-reaction ICP analysis of the filtrate rather than by yield retention alone.

### 3.3 Third, rational catalyst design needs a deeper mechanistic basis

*In situ* FTIR, *operando* XAS and DRIFTS could reveal the dynamic behaviour of Zr^4+^ and –SO_3_H sites during transesterification and allow defect concentration or pore size to be correlated with activation energy, replacing the empirical optimization that dominates the present literature.

### 3.4 Fourth, a full life cycle assessment is required

No study has compared the energy input, greenhouse gas emissions and overall environmental burden of MOF synthesis against conventional NaOH or H_2_SO_4_ processes. Without this, the environmental case for Zr-MOF catalysis rests on assumption rather than evidence, and the solvent-intensive nature of conventional MOF synthesis makes the outcome genuinely uncertain.

### 3.5 Fifth, the field must move to harder feedstocks

Most studies use refined oils or isolated model fatty acids, and as noted above only about a third address genuine triglyceride transesterification. Establishing a circular bioeconomy will require sustained work on microalgal lipids, non-edible *Jatropha* and *Ricinus* oils, and high-FFA waste frying oil, tested with the impurity profiles those feedstocks actually carry.

### 3.6 Sixth, photothermal and photocatalytic routes deserve attention as complementary strategies

He *et al.* have shown that a ternary S-scheme g-C_3_N_4_-TiO_2_@montmorillonite photocatalyst can drive esterification under illumination through microenvironment modulation,^104^ operating at far lower temperatures than the acid-catalyzed processes reviewed here. Such systems address a different limitation – energy input rather than active-site accessibility – and whether the Lewis acidity of Zr_6_ nodes can be coupled to a photothermal absorber is an open question worth testing. Likewise, Zhou *et al.* achieved 98.29% biodiesel yield from non-edible oils within 30 min under simulated solar irradiation using a sulfonated hydrochar catalyst whose interfacial localized photothermal effect exploits the full solar spectrum, including the near-infrared fraction.^105^

Translating Zr-MOF catalysts from laboratory to industry will require addressing these issues through hierarchical pore design, mixed-linker approaches, machine learning for framework screening, and continuous flow reactors. With further development, modified Zr-MOFs have the potential to become enabling materials for next-generation, low-carbon biodiesel production.

## 4 Conclusion

This review has compared five structural modification strategies for Zr-MOFs in biodiesel production rather than surveying them in isolation. Defect engineering activates an otherwise inert framework at minimal cost but confers no water or FFA tolerance; pore engineering relieves mass transfer limitations without adding active sites; linker functionalization with –NH_2_ and –SO_3_H groups gives the highest acid-site density and the only demonstrated water tolerance, at the expense of surface area; composite formation principally solves recovery and Brønsted acid supply; and enzyme immobilization permits the mildest conditions but the lowest yields.

Modification raises performance from 40% oleic acid conversion over conventionally synthesized UiO-66(Zr) to 97.3% over its amino-functionalized analogue under identical conditions, and to ∼98% FAME yield from waste oils over 5–8 cycles (Table 1 and Fig. 15). The decisive factor is not the choice of modification but how far a design resolves the trade-off between acid-site density and pore accessibility: every strategy that raises one tends to lower the other, and no reported system escapes the trade-off entirely—the most effective designs only mitigate it, by pairing hierarchical mesoporosity with grafted Brønsted acidity so that accessibility is partially preserved. Feedstock therefore determines the appropriate strategy more reliably than any general ranking.

Two limitations of literature qualify this conclusion. Cross comparison of yields is not meaningful, since studies differ in feedstock, alcohol and conditions, and normalization by catalyst-normalized productivity reverses the apparent ordering of several catalysts. And only about a third of the studies described as Zr-MOF biodiesel catalysis use genuine triglyceride feedstocks, so claims about biodiesel performance rest on a narrower base than the size of the literature suggests.

## Author contributions

Basem E. Keshta: conceptualization, writing – original draft, Project administration, methodology, investigation, writing – review & editing, formal analysis, data curation. Shweta Vyas, Lobna A. Heikal, Hany Koheil, Fatimah Bukola Shittu: writing – original draft, investigation, data curation. Eida S. Al-Farraj, Yasmeen G. Abou El-Reash, investigation, funding acquisition. Yuanbin Zhang: supervision, writing – review & editing.

## Conflicts of interest

The authors declare no conflict of interest.

## Data Availability

No primary research results, software, or code have been included, and no new data were generated or analyzed as part of this review. During preparation of this manuscript, Claude was used for English-language editing and to improve text clarity; no AI was used for data generation or analysis.
